# Cost effective technologies and renewable substrates for biosurfactants’ production

**DOI:** 10.3389/fmicb.2014.00697

**Published:** 2014-12-12

**Authors:** Ibrahim M. Banat, Surekha K. Satpute, Swaranjit S. Cameotra, Rajendra Patil, Narendra V. Nyayanit

**Affiliations:** ^1^Faculty of Life and Health Sciences, School of Biomedical Sciences, University of UlsterColeraine, UK; ^2^Center for Advanced Studies in Materials Science and Condensed Matter Physics, Department of Physics, Savitribai Phule Pune UniversityPune, India; ^3^Institute of Microbial TechnologyChandigarh, India; ^4^Department of Biotechnology, Savitribai Phule Pune UniversityPune, India; ^5^Department of Zoology, Sir Parashurambhau CollegePune, India

**Keywords:** biosurfactants, bioemulsifiers, fermentation, renewable, substrates, sustainable

## Abstract

Diverse types of microbial surface active amphiphilic molecules are produced by a range of microbial communities. The extraordinary properties of biosurfactant/bioemulsifier (BS/BE) as surface active products allows them to have key roles in various field of applications such as bioremediation, biodegradation, enhanced oil recovery, pharmaceutics, food processing among many others. This leads to a vast number of potential applications of these BS/BE in different industrial sectors. Despite the huge number of reports and patents describing BS and BE applications and advantages, commercialization of these compounds remain difficult, costly and to a large extent irregular. This is mainly due to the usage of chemically synthesized media for growing producing microorganism and in turn the production of preferred quality products. It is important to note that although a number of developments have taken place in the field of BS industries, large scale production remains economically challenging for many types of these products. This is mainly due to the huge monetary difference between the investment and achievable productivity from the commercial point of view. This review discusses low cost, renewable raw substrates, and fermentation technology in BS/BE production processes and their role in reducing the production cost.

## INTRODUCTION

Our daily routine basic activities are mostly dependent on the use of some kind of surfactants or emulsifiers including toothpaste, personal hygiene, cosmetic products, and other pharmaceutical by-products, most of which contains surfactants and emulsifier as one of their ingredients. The market for such products is therefore huge and demands are ever increasing. However, due to the non-biodegradability, ability to accumulate and toxicity of some of the chemical petroleum based product to the environment, there has been a general desire to find replacement surfactants to the chemically synthesized compounds with biological products (Satpute et al., 2010a,b; Marchant and Banat, 2012a,b). Such biological biosurfactant/bioemulsifiers (BS/BEs) are mainly of microbial origin and are generally more environmental friendly benign products.

Biosurfactants and bioemulsifiers amphiphilic surface active abilities which are due to the presence of hydrophobic and hydrophilic moieties within their molecules which allows them to aggregate at interfaces (between immiscible liquids, for example water and oil). BS/BE reduce surface and interfacial tension (IFT) in liquids or different phases of matter, like gas, liquid, and solid. Such properties play an important role in various fields like bioremediation, biodegradation, oil recovery, food, pharmaceutics, and many other applications in different industrial sectors (Cameotra and Makkar, 2004; Banat et al., 2010; Fracchia et al., 2014; Franzetti et al., 2014). The structural and functional novelty of such surface active molecules is attracting the attention of many researchers throughout the world. Their synthesis processes take place on water soluble and insoluble substrates by *de novo* pathway and/or assembly from other substrates (Satpute et al., 2010c).

The use of cheaper, renewable substrates from various industries such as agricultural (sugars, molasses, plant oils, oil wastes, starchy substances, lactic whey), distillery wastes, animal fat, oil industries have been reported and reviewed thoroughly by several researchers (Makkar et al., 2011). Various cheaper substrates such as soybean oil not only act as nutrients for the microbial growth but also act as an important source for isolation of potential BS producing microorganisms (Guerra-Santos et al., 1986; Lee et al., 2008). Rhamnolipids (RHL), one of the common BSs are usually produced on soybean oil soapstock, spent soybean oil, or chicken fat as a carbon source (Nitschke et al., 2004, 2005, 2010). Improvement in the fermentation technology, strain selection and use of cheaper, renewable substrates have a vital role in enhancing the production processes of BS industries (Marchant and Banat, 2012b; Marchant et al., 2014). However, large scale production for most microbial surface active agents has not reached a satisfactory economical level due to their low yields. In addition to this, high cost input is required for downstream processing to recover and purify microbial surfactants (Rodrigues et al., 2006a,b; Smyth et al., 2010a,b). Such obstacles may be overcome by isolating potential BS/BE producers that can use the renewable substrates to raise the quality as well as quantity of BS. We can make use of waste material as better substrates for BS/BE production. Several alternative strategies for production at commercial scale have been reviewed by Helmy et al. (2011).

This review aims to provide comprehensive information on various economical, renewable substrates that are used for production of BS and how these substrates have can support BS fermentation technology. We also give a brief glance on the kinetics of BS production and fermentation technology that has been improved since last two decades.

## NEED FOR CHEAPER, RENEWABLE SUBSTRATES IN BIOSURFACTANT INDUSTRY

Most biotechnological products processes need high monetary inputs and securing an optimum yield of product at the lowest expense through usage of low cost material (Cazetta et al., 2005). However, very low quantities of surface active agents are usually produced by microorganisms and the downstream processing of biotechnological products costs ~60–80% of the total production expenditure. This is why most of the marketable products based on BS and BE are quite expensive. Therefore, it essential to reduce the production costs of BS/BE through the use of inexpensive and renewable substrates (Desai and Banat, 1997; Banat et al., 2000; Makkar et al., 2011). A diversity of carbon (water soluble and water insoluble) and nitrogen sources have been used for BS production which may consequently vary in structure or production site within the cell (intra or extra cellular, cell associated) depending upon the substrate composition particularly the carbon source (Guerra-Santos et al., 1986; Fiechter, 1992). In addition to usual water soluble carbon sources, a variety of unusual carbon sources such as ethanol, blended gasoline, hydrocarbons like heptadecane, hexadecane etc. have been used (Shreve et al., 1995; Patricia and Jean-Claude, 1999; Prabhu and Phale, 2003; Cunha et al., 2004).

Increased public awareness of issues related to environmental pollution strongly influences the development of technologies that facilitates cleaning hazardous contaminants. This has given imputes for finding suitable cheap BS products that can be used in the treatment of such contaminations. Kapadia and Yagnik (2013) introduced an alternative approach using solid state fermentation to obtain a more economical viable production process worth implementing on a commercial scale. Some of the suggested strategies included the use of more cheaper materials, optimization of environmental conditions and screening for overproducing strain to attain the maximize productivity (Satpute et al., 2008). The efforts taken toward this direction are significant to claim BSs as the molecules of the future. It is important to note that the results obtained to date show encouraging potentials to drive a beginning for the BS production industry.

## USE OF COST EFFECTIVE RENEWABLE SUBSTRATES FOR BIOSURFACTANT PRODUCTION

Different relatively cheap and abundant substrates are currently available for use as carbon sources from various industrial sectors (**Table 1**). Many of these substrates have been reported as suitable substrates for growth and production of a wide range of microbial amphiphilic molecules (see **Tables Tables 2** and **3**). These substrates are described in detail as follows.

**Table 1:** Summary of various cheaper/renewable substrates available from different industrial sectors.

Source industry	Waste/residues as *cheaper, renewable substrate*
Agro-industrial waste, crops residues	*Bran, beet molasses, Bagasse of* sugarcane straw of wheat, cassava, cassava flour wastewater, rice straw of rice, hull of soy, corn, sugar cane molasses
Animal fat	Waste
Coffee processing residues	Coffee pulp, coffee husks, spent of free groundnut
Crops	Cassava, potato, sweet potato, soybean, sweet sugar beet, sorghum
Dairy industry	Curd whey, cheese whey, whey waste
Distillery industry	Industrial effluents
Food processing industry	Frying edible oils and fats, olive oil, potato peels rape seed oil, sunflower, vegetable oils
Fruit processing industry	Banana waste Pomace of apple and grape, carrot industrial waste, pine apple
Oil processing mills	Coconut cake, canola meal, olive oil mill waste water, palm oil mill, peanut cake, eﬄuent, soybean cake, soapstock, waste from lubricating oil

**Table 2:** Summary of various renewable substrates used for production of microbial amphiphilic molecules by *Acinetobacter*, *Bacillus,* and *Candida* sp.

Organism	Renewable substrate	Biosurfactant/bioemulsifier type	Reference
*Acinetobacter*	Renewable resources	Surface active polymers	Rosenberg and Ron (1998)
*Acinetobacter calcoaceticus*	Soap stock oil (SSO)	Expolysaccharide	Shabtai (1990)
*Bacillus subtilis*	Molasses	Surfactin	Makkar and Cameotra (1997)
*B. subtilis* ATCC 21332; *B. subtilis* LB5	Cassava flour wastewater	Lipopeptide	Nitschke and Pastore (2004, 2006)	
*B. subtilis*	Potato cassava	Surfactin	Noah et al. (2002)
*B. subtilis*	Potato cassava	Surfactin	Noah et al. (2005)
*B. subtilis*	Potato waste	Surfactin	Thompson et al. (2000)
*B. subtilis*	Potato waste	Surfactin	Thompson et al. (2001)
*Bacillus* sp.	Lubricating oil	Lipopeptide	Mercadé et al. (1996)
*Bacillus subtilis* ATCC 21332	Potato waste	Surfactin	Fox and Bala (2000)
*B. subtilis*	Peat hydrolysate	Surfactin	Sheppard and Mulligan (1987)
*B. subtilis* NB22	Solid state fermentation	Peptide antibiotic iturin	Ohno et al. (1993)
*B. subtilis* (recombinant)	Solid state fermentation	Lipopeptide antibiotic surfactin	Ohno et al. (1995)
*B. subtilis* NB22 (recombinant)	Wheat bran	Lipopetide-surfactin	Ohno et al. (1992)
*Candida antarctica*, *C. apicola*	Oil refinery waste	Glycolipids	Deshpande and Daniels (1995)
*C. bombicola*	Animal fat	Sophorolipid	Deshpande and Daniels (1995)
*C. bombicola* ATCC 22214	Turkish corn oil and honey	Sophorolipids	Pekin et al. (2005)
*C. lipolytica* 1055 and 1120	Babacu oil	Bioemulsifier	Sarubbo et al. (1997)
*C. lipolytica* IA1055	Babassu oil	New biemulsifier: carbohydrate, lipid, protein	Vance-Harrop et al. (2003)
*C. bombicola*	Soy molasses-based medium	Sophorolipids	Solaiman et al. (2004, 2007)
*C. bombicola* ATCC 22214	Whey and rapeseed oil	Sophorolipid	Daniel et al. (1998a,b)
*C. bombicola*	Canola oil	Biosurfactant	Zhou and Kosaric (1995)
*C. lipolytica*	Industrial residue	Biosurfactant	Rufino et al. (2007)
*C. lipolytica*	Canola oil	Biosurfactant	Sarubbo et al. (2007)
*Candida* sp. SY16 95 45	Soybean oil	Mannosylerythritol lipid	Kim et al. (2006)
Yeast	Oil refinery waste	Glycolipids	Bednarski et al. (2004)

**Table 3:** Summary of various renewable substrates used for production of microbial amphiphilic molecules by *Pseudomonas* and other strains.

Organism	Renewable substrate	Biosurfactant/bioemulsifier type	Reference
*Cladosporium resinae*	Jet fuel JP8	Biosurfactant	Muriel et al. (1996)
*Corynebacterium kutscheri*	Waste motor lubricant oil and peanut oil cake	Biosurfactant	Thavasi et al. (2007)
*Peudomonas cepacia*	Sunflower oil	Bioemulsifier	Fiebig et al. (1997)
*P. aeruginosa* LB1	Oil wastes	Rhamnolipid	Nitschke et al. (2005)
*P*. *aeruginosa*	Whey	Rhamnolipid	Koch et al. (1988)
*P. aeruginosa*	Molasses	Rhamnolipid	Raza et al. (2007a)
*P. aeruginosa* AT10	Soybean oil refinery wastes	Rhamnolipid	Abalos et al. (2001)
*P. aeruginosa* GS9-119 *P. aeruginosa* DS10-129	Sunflower and soybean oil	Rhamnolipid	Rahman et al. (2002)
*P. aeruginosa* GS3	Molasses	Rhamnolipid	Patel and Desai (1997)
*P. aeruginosa* strain BS2	Distillery and whey waste	Rhamnolipid	Dubey and Juwarkar (2001)
*P. aeruginosa* strain BS2	Distillery and curd whey wastes	Rhamnolipid	Babu et al. (1996)
*P. aeruginosa* strain BS2	Curd whey and distillery waste	Rhamnolipid	Dubey and Juwarkar (2004)
*P. aeruginosa* strain BS2	Fermented distillery wastewater	Rhamnolipid	Dubey et al. (2005)
*P. aeruginosa* strain LBI	LB1 soapstock	Rhamnolipid	Benincasa et al. (2002)
*P. aeruginosa* strain LBI	LB1 soapstock	Rhamnolipid	Benincasa et al. (2004)
*Pseudomoas* sp. DSM 2874	Rapeseed oil	Mixture of four types of glycolipids (rhamnolipid 1–4), L-(+)-rhamnose and (R, R)-3-(3-hydroxydecanoyloxy) decanoic acid	Trummler et al. (2003)
*Pseudomonas* sp.	Jet fuel JP8, diesel oil	Biosurfactant	Bento and Gaylarde (1996)
*Pseudomonas* sp.	Used olive, sunflower oil	Rhamnolipid	Haba et al. (2000)
*P. aeruginosa*	Vegetable oil refinery wastes	Biosurfactant	Raza et al. (2007b)
*P. aeruginosa* FR	Palm oil	Biosurfactants	Oliveira et al. (2006)
*Pseudomonas* sp. JAMM	Olive oil mill eﬄuent (OOME)	Rhamnolipids	Mercadé et al. (1993)
*Rhodococcus* sp.	Waste lubricating oil	Trehalose glycolipids	Mercadé et al. (1996)
*Trichosporon montevideense* CLOA 72	Dairy industry effluents	Glycolipid	Monteiro et al. (2009)
*Tsukamurella* sp. DSM 44370	Natural vegetable oil	Glycolipid	Vollbrecht et al. (1999)

### AGRO-INDUSTRIAL WASTE, CROPS RESIDUES

Products such as bran, straw of wheat, straw of rice, hull of soy, corn, rice, sugar cane molasses, beet molasses, bagasse of sugarcane, cassava flour and its wastewater are representative candidates of agro-industrial waste (Nitschke et al., 2004; Rashedi et al., 2005a; Benincasa, 2007; Thavasi et al., 2014). Some waste material like rice water (by-product from domestic cooking and rice processing industry), corn steep liquor (corn processing industry) and cereals, pulses processed waste water are rich in starch content. Agro-industrial waste contains high amount of carbohydrates, lipids and hence, can be used as a rich carbon source for microbial growth. Among the agro-industrial waste products, molasses had attracted considerable attention by many researchers.

Molasses are concentrated syrups by-products of sugar cane and beet processing industries. This cheap substrate contains 75% dry matter, 9–12% non-sugar organic matter, 2.5% protein, 1.5–5.0% potassium and ≈1% calcium, magnesium, and phosphorus. Other components like biotin, pantothenic acid, inositol, and thiamine at 1–3% are also present giving it a thick, dark brown colored appearance. The high sugar content ranging approximately between 48 and 56% represents a good substrate for growth as well as production of microbial bioactive compounds for various microorganisms. When molasses are used as substrate, it needs to be clarified otherwise some of the components from molasses itself may impart unfavorable color to the desired products reducing their quality (Raza et al., 2007a,b). Molasses clarification process, however, can be quite costly as it involves dilution with water, acidification, pH adjustment to 7.0 using CaO powder and addition of K_4_Fe(CN)_6_ as a coagulant is carried out. During this process, heating up to 90°C for 1 h and cooling overnight at room temperature permits settlement of suspended solids and fibrous particles which can be removed by centrifugation (Raza et al., 2007a,b).

Achieving cost effective BS production depends on the development of cheaper processes and the provision of low cost substrate raw material. Most earlier research concentrated on *Pseudomonas* sp. and *Bacillus* species while using molasses, whey, CSL as carbon and energy sources (Makkar and Cameotra, 1997, 1999; Patel and Desai, 1997; Makkar et al., 2011). However, there is a threat that the commercial products may get contaminated with those cheaper substrates products that are used as raw materials for production process. When the pure products are not available, there is difficulty to use them for intended application purposes. Various industrially important products like citric acid, xanthan gum, baker’s yeast, acetone, alcohol, vitamins, amino acids, and organic acids are also produced successfully using molasses as a substrate.

India has an economy dependent on agro industries producing large volume of agro-industrial wastes which are mostly suitable for use as substrate. Some of the research laboratories are particularly involved in the use of molasses for production of various microbial metabolites. Makkar and Cameotra (1997) reported BS production from two *Bacillus subtilis* cultures in minimal medium supplemented with molasses as carbon source. Optimum BS production with good emulsification activity (EA) was achieved in late stationary phase. Patel and Desai (1997) also worked on production of RHL BS from *Pseudomonas aeruginosa* GS3 by using molasses and corn steep liquor. Molasses of 7% (v/v) and corn steep liquor of 0.5% (v/v) were appropriate for optimum BS production. Cells produced BS in the stationary phase with a rhamnose sugar concentration of 0.24 g/L. Joshi et al. (2008) also used molasses and other carbon sources to produce BSs from several *Bacillus* strains under thermophilic conditions.

At international level several researchers have contributed in this area. Raza et al. (2007a) produced RHL BS from *P*. *aeruginosa* mutant strains using blackstrap molasses with or without supplementary nitrogen source and reported a yield of 1.45 g/L RHL after 96 h incubation. Another interesting work contributed by Benincasa (2007) suggests that the RHL produced from agro industrial wastes has an important role for hydrocarbon biodegradation in contaminated soil. Such studies have proved the importance of agro industrial wastes in bioremediation processes. Onbasli and Aslim (2009) used sugar beet molasses for RHL BS production from *Pseudomonas* strains and showed that among 18 strains of *Pseudomonas*, *P*. *luteola* B17 and *P. putida* B12, gave high yield of RHL at 5% (w/v) molasses. Cultures isolated from oily sites also utilize sugar beet molasses effectively for BS production. Rashedi et al. (2006), reported RHL BS producing *P. aeruginosa* isolated from Iranian oil wells. They used waste dates as sole carbon for the production of RHL using fed-batch culture and reported improved yields of BS. It is important to note that yield of the BS production increases with the increased concentration of molasses; maximum production, however, was reported using a medium containing 7% (v/v) of molasses. Other than above mentioned sources of molasses (sugar cane and beet), soy molasses are the most commonly used wastes from industrial sectors in the production of sophorolipid (SL) type BSs (Deshpande and Daniels, 1995; Solaiman et al., 2007). Molasses produced during the production process of soybean oil have been reported as a good carbon sources for SLs type BS from *Candida bombicola* (Solaiman et al., 2004). About 21 g/L yield was obtained as compared with glucose and oleic acid (79 g/L) in fermentation process. Such studies may not show benefits in enhancing the yield of metabolite but may be useful ways to reduce the accumulation of waste disposals from oil industry.

Researchers have worked with various combinations of carbon and nitrogen sources in BS production technology. Joshi et al. (2008) used molasses along with cheese whey as substrate for BS production from *Bacillus* sp. At the temperature of 45°C, the strain shows maximum BS production using molasses at 5.0–7.0% (w/v). Similar reports on BSs produced from probiotic bacteria have also been described. Rodrigues et al. (2006b) carried out studies with two microbial cultures namely, *Lactococcus lactis* 53 and *Streptococcus thermophilus* for BS production with conventional synthetic medium. They reported maximum BS production of 0.8 g/L for *S*. *thermophilus* and 0.7 g/L for *L. lactis* 53. Molasses have been found to enhance the yield of BS when compared to other conventional synthetic media. Thus, authors have suggested that there is not only an increase in the (about 1.2–1.5 times) mass of BS per gram of cell dry weight but also about 60–80% reduction in medium preparation costs. Therefore, molasses has been proved as an alternative economical medium for commercial BS production processes.

Cost variation in commercial production process has been calculated for molasses and soybean oil used as substrates (Joshi et al., 2008). Although this work is reported long time back, it showed that the substrate alone would place the BS production cost at a competitive setting when compared to chemical surfactants such as alcohol ethoxylate and alkylphenoletoxylate types for use in enhanced oil recovery (EOR). The point here to be highlighted is when molasses are used as a substrate BS production becomes more expensive than the chemically synthesized surfactant. On the other hand, the sophorose lipids employed in cosmetics is valuable as compared to the use of the synthetic surfactant, for the same cosmetic application. Usually various agricultural wastes like barley bran, trimming vine shoots, corn cobs, and *Eucalyptus globulus* chips have been used for simultaneous lactic acid and BS production. *Lactobacillus pentosus* has been tried in BS fermentation process by using hemicellulosic hydrolyzates after nutrient supplementation. The highest value of reduction (21.3 units) was found when using hemicellulosic sugar hydrolyzates obtained from trimming vine shoots, that corresponds to 0.71 g of BS per gram of biomass and 25.6 g of lactic acid/L. Whereas, barley bran husk hydrolyzates produces 0.28 g of BS per g of biomass and 33.2 g of lactic acid/L (Moldes et al., 2007).

### ANIMAL FAT AND OIL INDUSTRIES AS SUBSTRATES

**FIGURE 1:**
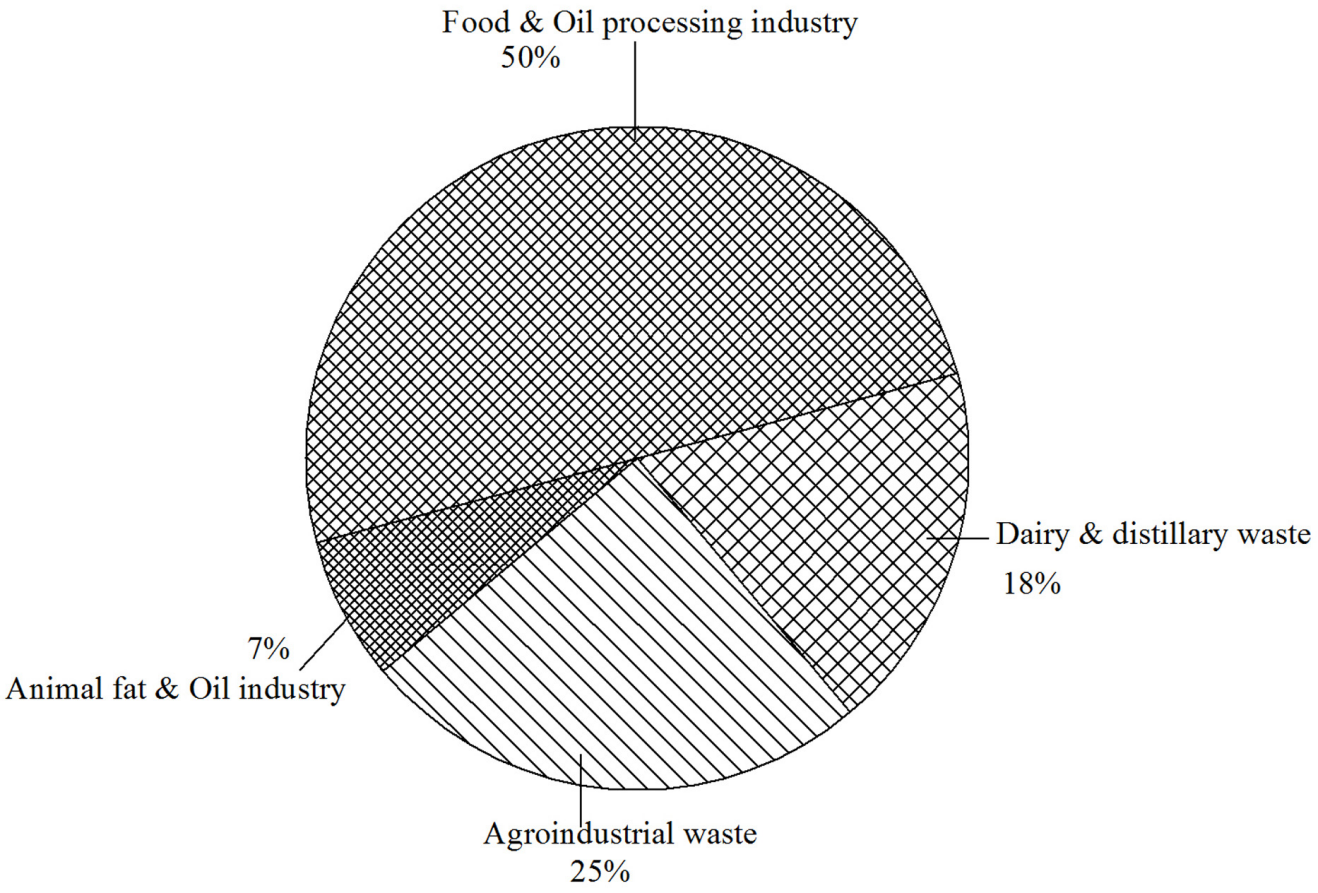
**Approximate percentage distribution for literature available on various renewable substrates used for biosurfactants production**.

Meat processing industries such as food and leather produces significant quantities of animal fat, tallow and lard. Demand for animal fats is considerably less than vegetable oils and much of it becomes a problem for utilization as well as for their disposal. In comparison with other renewable substrates, animal fat and oil has not been much explored (**Figure 1**). An alternative option for such products is using them as raw material or substrates for production of commercial imperative compounds. Animal fat has been reported to act as a stimulator for the production of SLs BS from *C. bombicola* yeast (Deshpande and Daniels, 1995). One of the main outcome of their investigation indicated that this yeast grows poorly in presence of fat alone in the production medium. Mixture of glucose (10% w/v) and fat (10% v/v), however, enhances the growth of the yeast and the production of SLs (120 g/L). Recently, Santos et al. (2013) reported maximum glycolipid BS production using animal fat combined with corn steep liquor as compared to other carbon sources using yeast *Candida lipolytica* UCP0988. They also reported the product to have uses in bioremediation, oil mobilization, and recovery.

Production of BSs by fermentation of fats, oils, and their co-products has also been reported (Solaiman et al., 2003). Nitschke et al. (2010) carried out BS production by using soybean oil waste (manipueira), along with molasses, whey and cassava flour, as substrates. Their observation suggests that cassava flour wastewater as a promising source of nutrients for BS production. These cheaper substrates were compared with conventional medium for BS production. Among eleven isolates tested, eight cultures reduced the surface tension (SFT) to levels below 30 mN/m using manipueira as substrate. They reported improved growth on manipueira agar for several isolates suggesting a high growth capacity and concluded that manipueira represents a potential alternative culture medium for BS production.

Industrial wastes, corn steep liquor and ground-nut oil refinery residue were also reported as low cost nutrients for the production of glycolipid type BS from *Candida sphaerica* (UCP 0995). The strain successfully mobilizes and recovers about 95% of motor oil adsorbed on sand sample which has vast applications in bioremediation processes (Luna et al., 2012). Several contributions are reported on usage of refinery wastes for production of microbial products. *Trichosporon mycotoxinivorans* CLA2 is BE-producing yeast strain which was isolated from the dairy industry effluents on mineral minimal medium containing refinery waste. This refinery wastes consists of diatomaceous earth impregnated with esters of having high organic matter content. Like molasses, pre-treatment of refinery waste is therefore, necessary for subsequent disposal in to the environment. Very few types of BEs have been produced from these residues (Monteiro et al., 2012).

### DAIRY AND DISTILLERY INDUSTRIES BY-PRODUCTS

Dairy industries produce large quantities of whey that includes, curd whey, whey waste, cheese whey, lactic whey, all of which are easily available as raw substrate for microbial production of metabolites (Dubey and Juwarkar, 2001, 2004; Makkar and Cameotra, 2002; Dubey et al., 2005; Rodrigues and Teixeira, 2008). High amount (about 75%) of lactose is present in the lactic whey. Other components like protein and organic acids, vitamins provide good sources for microbial growth and BS production (Maneerat, 2005). Interesting studies have been reported regarding cloning the gene Lac ZY for lactose utilizing capability from *Escherichia coli* into *P*. *aeruginosa*. The cloned strain of *P*. *aeruginosa* grew well on whey and produces some RHL (Koch et al., 1988). Kosaric et al. (1984) have suggested the multi-organism strategy to decrease the cost at the commercial scale.

**FIGURE 2:**
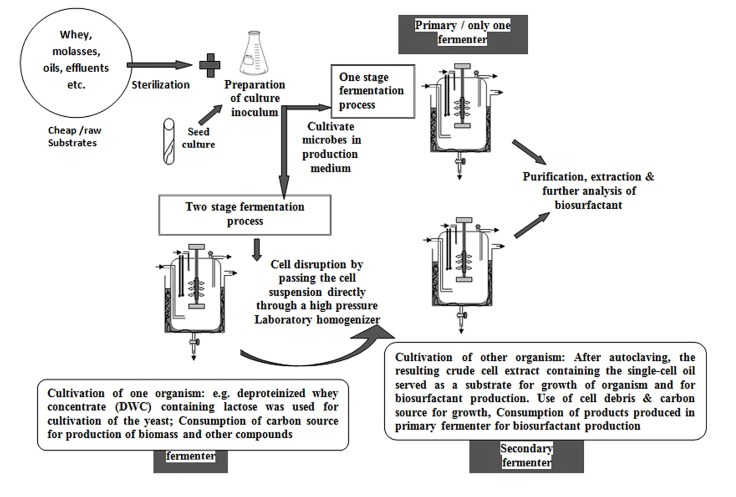
**Pictorial representation for one stage and two stage fermentation process for biosurfactant production.** Several authors have used these approaches. We have constructed figure based on Daniel et al. (1999) experimental work.

Other than RHL, a glycolipid type BS, considerable work has been reported on SLs. SLs has been produced by a two-stage (**Figure 2**) process starting from deproteinized whey concentrate (DWC) by using *Cryptococcus curvatus* ATCC 20509 and *C. bombicola* ATCC 22214 (Otto et al., 1999). These researchers compared the products from one-stage processes, by using different lipid based substrates. Two-stage batch cultivation process suggested that various physicochemical and properties of the SLs are greatly influenced by different carbon sources and not by the cultivation conditions. The same research group (Daniel et al., 1998b) had worked on the strains mentioned above using whey concentrates alone and in combination with rapeseed oil for production of SLs using single step batch cultivation. They developed sterilization method for whey by a combination of cross flow and sterile filtration. *C. bombicola* ATCC 22214 produced high (280 g/L) yield of SLs. Surprisingly, Daniel et al. (1998b) reported that *C. bombicola* ATCC 22214 does not consume whey lactose while it grows on oil or the lipidic substrates for SLs production. Daniel et al. (1998a) had also worked on two-stage batch cultivation concept reporting high yields (422 g/L) of SLs production using substrates like whey concentrate and rapeseed oil. The group also had grown the oleaginous yeast *C. curvatus* ATCC 20509 on DWCs in the first stage where they noted that lactose was consumed completely and biomass as well as an intracellular triglyceride, so-called single-cell oil (SCO), were produced. Crude cell extract resulted from cell disruption and heat sterilization were used for growth as well as SLs production by the yeast *C. bombicola* in a second stage (Daniel et al., 1999). The authors also showed that starting from DWC (50 g/L lactose), in the two stage fermentation process resulted in 12 g/L of extracellular SLs. In this two stage type of process, they reported that neither the growth of *C. bombicola* nor the productivity of SL from SCO is affected by the concentration of whey. In spite the amount of oil was the overall limiting factor of the process. Such kind of problems can be overcome by the addition of cheap oils during the production phase, to allow achieving high yields of SLs.

Wastewaters generated by dairy industries contain large quantities of fats and oils which are to some extent difficult to degrade (Willey, 2001; Cammarota and Freire, 2006). Like molasses, refinery wastes, pretreatment or clarification process for wastewater is a costly process. Daverey et al. (2009) described a process to utilize such valuable bioproducts for SLs production using yeasts *C. bombicola* through supplementing with sugarcane molasses and soybean oil. They reported about 38.76 g/L of SL with the synthetic dairy wastewaters containing 50 g/L sugarcane and 50 g/L soybean oil. Thus, authors therefore suggested utilizing real dairy industry wastewater for both the production of SLs. In addition to the above reports, Babu et al. (1996) reported on RHL production batch kinetic using distillery and whey waste in comparison to synthetic medium. Both the specific growth rates (μmax) and specific product formation rates (Vmax) were comparatively better in both waste media than in the synthetic media. Thus, their studies have proved that industrial wastes from distillery and whey are resourceful substrates for BS production. Similar observations were reported by Babu et al. (1996) and Dubey and Juwarkar (2001) for RHL production from *P. aeruginosa* by utilizing distillery eﬄuent and whey wastes. Dubey and Juwarkar (2004) demonstrated 0.91 and 0.92 g/L of BS and use of *P*. *aeruginosa* strain in reduction of the pollution load up to 85–90%.

Although using dairy and distillery waste for various BS productions is possible, difficulties may arise when attempting to purify or collect such products. BSs recovery methods are diverse and include solvent extraction, precipitation, crystallization, centrifugation, and foam fractionation (Satpute et al., 2010a). However, Dubey and Juwarkar (2004) suggested that the various purification mentioned above cannot be effectively employed when using distillery wastewater as a nutrient medium for BS production. They produced BSs using *P*. *aeruginosa* strain BS2 and distillery wastewater and suggested that the substrate imparted color to the produced products which had a non-esthetic appearance and was difficult to recover from the fermentation medium. Therefore, they extended a new downstream technique involving adsorption-desorption processes using wood-based activated carbon (WAC). Therefore, WAC is one of the most efficient adsorbent among adsorbing materials like silica gel, activated alumina and zeolite. Polar solvent like acetone were also found to be efficient in recovering up to 89% BS from WAC. The authors recommended that WAC can be reused for BS recovery up to three cycles. The contribution by Dubey and Juwarkar (2004) has provided new approach for continuous recovery of BS from fermented distillery waste and concentrated foam. Such techniques can reduce the cost involved in the solvent based purification methodology at the same time as providing efficient yield.

The use of distillery and whey waste as substrates for production of BSs is a useful recycling and reuse process. Babu et al. (1996) established maximum specific growth rates, specific product formation rates and proved to be superior to the synthetic medium. The provision of substrates from such wastes represents a huge contribution to future BS production industries. An exciting report has been published by Dubey et al. (2012) on distillery waste in combinations with curd whey waste, fruit processing waste and sugar industry eﬄuent for growth and production of BS from newer microbes. They observed a positive impact of such combinations for BS production from *Kocuria turfanesis* strain BS-J and *P. aeruginosa* strain BS-P. The authors have suggested that we can replace precious water with other wastes required for diluting distillery waste for BS production. Instead of fruit processing waste, fruit juices like pineapple juice are also becoming attractive alternative carbon sources. Govindammal and Parthasarathi (2013a) carried out this studies and demonstrated 9.43 g/L yield from *P. fluorescens* MFS03 isolated from the crude oil enriched mangrove soil to improve the process economics.

### OIL PROCESSING INDUSTRIES

Wastes from oil processing industries represent one of the alternative and easily available renewable substrates for production of microbial surface active molecules. Few examples are listed below. It is important to note that, the vegetable oil is one of the first substrates reported for high yield of SLs from *Torulopsis bombicola*. A SLs with the yield of 67 g/L has been reported by Cooper and Paddock (1984). Not only, sophorose lipid type BS but also other types of glycolipids have been studied extensively by using wastes from oil industries. Robert et al. (1989) and Mercadé et al. (1993) disclosed that the usage of vegetable oil from the distillation process and is effective for RHL production from *Pseudomonas* strains. Various oils along with water soluble carbon sources are proved to be good substrates for microbial surfactant molecules. This is evident from the following example. Babacu oil (5% v/v) supplemented with glucose (1% w/v) as carbon source provides a good source for growth and BE production. This work carried out by Sarubbo et al. (1999) suggested that two strains of *C. lipolytica* (1055 and 1120) produce BEs at the end of the exponential growth phase and beginning of the stationary growth phase.

Olive oil, sunflower has been proved as potential carbon and energy sources for production of microbial surfactants. The oils that contain low chain length (<C10) fatty acids undergoes modification for incorporation into surface active products. Haba et al. (2000) investigated the use of olive, sunflower oils in submerged culture condition by 36 microbial strains. They reported that several *Pseudomonas* strains usually grows well on waste olive or sunflower oil (2%) reducing SFT (<40 mN/m) of production medium while *Bacillus* strains do not use these substrates efficiently. Other strains like *Rhodococcus*, *Acinetobacter calcoaceticus,* and *Candida* neither use oils for growth nor for BS production. Abalos et al. (2001) also used a soybean oil refinery waste for production of RHLs using *P. aeruginosa* AT 10 strain and detected seven different homologs (R_2_C_10_C_10_ + R_1_C_10_C_10_ + R_2_C_10_C_12_ + R_1_C_10_C_12_ + R_1_C_12_:1C_10_ + R_1_C_12_:_2_ + R_1_C_8_:_2_) totaling ≈9.5 g/L. They also reported excellent antifungal properties against various filamentous fungi. A range of saccharic and lipidic feed stoke has been frequently used to produce SLs using *C. bombicola*. The fatty acid unsaturation, carbon chain length and source of low-cost industrial lipid feed-stocks influenced SLs production (Felse et al., 2007).

Rahman et al. (2002) used soybean oil, safflower oil and glycerol for production of RHLs using cultures of *P. aeruginosa*. Soybean oil supplements helps in increasing the biomass and RHL production to several fold that obtained just with safflower oil and glycerol. Increased amount of SL type BS has been produced by increasing the concentrations of safflower oil and glucose. Further yield can be enhanced with increased concentration of yeast extracts (Zhou et al., 1992). Use of such low cost renewable substrates in BS fermentation technology could be applied for bioremediation of hydrocarbon-contaminated sites and oil recovery process.

Among the above mentioned substrates, glycerol represents an important renewable carbon source as it is one of the main by-product of the biodiesel, biofuel production processes worldwide. For example, 1 kg of glycerol is generated from 10 kg of bio-diesel when rapeseed oil is used (Meesters et al., 1996). Decanoic acid and rapeseed oil were used by Trummler et al. (2003) to grow *Pseudomoas* sp. DSM 2874 and produce mixture of four types of glycolipids (rhamnolipid 1–4), L-(+)-rhamnose and (R, R)-3-(3-hydroxydecanoyloxy). Fed-batch process with rapeseed oil produced mixtures of mono and di RHLs at a very high yield of 45 g l^-1^. Another important work has been reported by Thanomsub et al. (2004) on glycolipid monoacylglycerols BS from *Candida ishiwadae*. This strain was isolated from plant material in Thailand on soybean cooking oil. Yeasts such as *C. bombicola* ATCC 22214 also efficiently used corn oil and honey for SLs production achieving higher yields when both grown on sugar and oil (Pekin et al., 2005).

In addition, Abouseoud et al. (2007) achieved BS production from *P. fluorescens* Migula 1895-DSMZ using olive oil as a carbon source with ammonium nitrate as a nitrogen sources. The products is reportedly a type of glycolipid with various properties like foaming, emulsifying and antimicrobial activities in addition to being highly stable at 120°C for 15 min, NaCl (10% w/v) and a wide range of pH values. *Tsukamurella* sp. DSM 44370 also used vegetable oil for its growth in addition to glycolipid BS production. Mutant strains of *P. aeruginosa* EBN-8 produced BS on canola, soybean, and corn oil refineries (Raza et al., 2007a,b). Canola oil refinery waste supplemented with sodium nitrate was reported best for microbial growth and RHL production with a yield of 8.50 g/L. Co-utilization of canola oil and glucose has also been carried out successfully for production of BS from *C. lipolytica* (Sarubbo et al., 2007). Oil wastes from cottonseed, soybean, palm oil, babassu, and corn oil refinery were studied as substituting low-cost substrates for RHL production by *P*. *aeruginosa* LBI strain. Marine microbial strains can also make use of oils (e.g., olive oil) other than aromatic hydrocarbons or crude oil for BS production. Khopade et al. (2012) reported potential BS producing strain, marine *Nocardiopsis* B4 isolated from the west coast of India. The BS is stable at higher temperature (100°C), wide range of pH and salt concentrations. Olive oil has been proved to enhanced BS production. In some cases use of only pure carbon sources may not give the high yield of BS. However, disaccharides like lactose if supplemented with olive oil, the prominent difference can be seen in the intra- and extracellular lipids synthesized by the microbes. This concept has been showed from the work by Zhou and Kosaric (1993).

Palm oil mill eﬄuent is also a promising substrate for BS production. Palm oil has also been used for BS production using *P. aeruginosa* SP4 (Pansiripata et al., 2010). A newly isolated BS-producing strain namely *Nevskia ramosa* NA3 has been reported for production of 1.0 g/L BS on palm oil mill eﬄuent (Chooklin et al., 2013). Saimmai et al. (2012) also documented BS and BE producing microorganisms from palm oil contaminated industrial sites in palm oil refinery factory. Along with palm oil, they also included other sources like palm oil decanter cake and palm oil mill eﬄuent. Use of such kind of different oil for screening and BS production process has successfully resulted five new genera namely, *Buttiauxella*, *Comamonas*, *Halobacterium*, *Haloplanus,* and *Sinorhizobium* for the first time. Such studies are significant for the future development of economically efficient industrial-scale biotechnological processes. Studies by Thaniyavarn et al. (2008) indicated the SL production by *Pichia anomala* PY1, a thermo tolerant strain isolated from fermented food. They used 4% soybean oil as carbon source at pH 5.5 and 30°C for 7 days. Comparative studies on media supplemented with both glucose or soybean oil lead to good BS production.

Comparative studies carried out by Govindammal and Parthasarathi (2013b) on glucose, petroleum based substrates, waste fried vegetable oil, and coconut oil cake for BS production from *Pseudomonas fluorescence* MFS03 isolated from mangrove forest soil. They proved that vegetable oil and coconut oil are reliable substrates for BS production. These oils contain high percentage of oleic acid.

Very recently, Saravanan and Subramaniyan (2014) isolated *P. aeruginosa* PB3A strain from oil contaminated soil and examined BS production on various substrates namely, castor oil, coconut oil, rapeseed oil corn oil, motor oil, sunflower oil, olive oil, olein, barley bran, cassava flour waste, rice bran peanut cake, potato waste, and wheat bran instead of routine carbon sources. Corn oil and cassava waste flour were found to be highly effective. Once again these studies have confirmed the potential role of agro-industrial wastes for BS production in place of synthetic media.

Sometimes the oils in the production medium needs to be supplied along with other ingredients like mineral salts, glucose. Bento and Gaylarde (1996) carried out BS production from *Pseudomonas* by growing in the production medium with sterile diesel oil, mineral salts, and glucose. Other oil sources like jet fuel JP8 also act as rich carbon source. Muriel et al. (1996) worked on Jet fuel JP8 for BS production from *Cladosporium resinae* where SFT of the production medium was lowered significantly with the increase in emulsion and foaming properties. Thavasi et al. (2009) reported *Azotobacter chroococcum* a BS producing strain isolated from marine environment able to grow on waste motor lubricant oil, crude oil, and peanut oil cake. Peanut oil cake was reported as best source for BS production with a yield of 4.6 g/L and an ability to emulsify various hydrocarbons effectively. Studies from Thavasi et al. (2007) described the outlook for BS production by using relatively cheap and abundantly available resources such as peanut oil cake and waste motor lubricant oil. This fact is supported from the studies reported on production and characterization of glycolipopeptide BS from *Corynebacterium kutscheri*. Studies showed optimum growth (9.8 g/L) and BS production (6.4 mg/ml) in fermentation medium with peanut oil cake. This glycolipopeptide emulsifies crude oil, waste motor lubricant oil, kerosene, diesel, peanut oil, xylene, naphthalene, and anthracene which have applications in various hydrocarbons in bioremediation processes. This study has proved potential role of BS in bioremediation process. Peanut oil has been used by probiotic bacterial system (*Lactobacillus delbrueckii*) for production of BS (Thavasi et al., 2011).

In addition to these inexpensive sources, spent yeast from fermentation industries has also been utilized in the production of high value product from a commercial point of view (Alcantara et al., 2012). Vance-Harrop et al. (2003) used babassu oil and D-glucose as carbon sources for the BS production from yeast strain *C. lipolytica* IA1055. This BS is composed of carbohydrate, lipid, protein in production medium prepared in natural seawater (diluted up to 50% v/v) supplemented with urea, ammonium sulfate, and phosphate. Most literature suggests exploitation of natural processes and developing economically viable production of BSs through the use of oil industry wastes. Bhardwaj et al. (2013) recently reviewed the production and applications of BSs from the oleo-chemical industrial wastes. Waste oils can be used for screening and selection of microbes for their waste oil utilizing capacity and BS production. Mercadé et al. (1996) carried out such studies where, lube oil was used to study 44 different cultures isolated from hydrocarbon-contaminated soil samples. Their studies showed that about 10% of the strains isolated shows BS production. These strains include *Rhodococcus* sp. for trehalose glycolipids and *Bacillus* sp. for lipopeptide type BS synthesis.

Spent oils are usually abundantly available oils that are quite difficult to dispose of due to environmental concerns including persistence and resistance to biodegradation (Mercadé et al., 1996). They include waste vegetable oil, used motor oil, lubricating oils, jet fuels all of which can act as cheaper source for microbial processes such as BS production. Usage of such kind substrates is usually encouraged as a pollution control strategies. Food processing industries use huge quantity of frying oils, where the composition vary depending on the number of times it has been used, modification in its composition, and finally need for pretreatment.

Studies carried out by Morita et al. (2007) on production of glycolipids by basidiomycete yeast *Pseudozyma antarctica*, on glycerol with the yield of 0.3 g/L of a BS. Another contribution on this aspect is shared by Ashby et al. (2005), where they used 40% of glycerol and 34% of hexadecane soluble compounds (92% of fatty acids and 6% of monoacylglycerol/triacylglycerol) and 26% of water for SLs synthesis by *C. bombicola*. About 60 g/L yields of SL was obtained from these studies. Several reports in literature support the use of glycerol as a carbon source for BS production (Guerra-Santos et al., 1986; Santa Anna et al., 2002; Rashedi et al., 2005b). Not only *Candida* and/or *Pseudomonas* spp. utilize glycerol as carbon source. Fontes et al. (2012) reported a wild type *Yarrowia lipolytica* for BS production using residual glycerol or clarified cashew apple juice present abundantly in Brazil. High amount of olive oil mill wastewater is produced from the olive oil extraction procedures. Olive oil mill eﬄuent (OOME) appears as a concentrated black color liquor that contains water-soluble polyphenols which usually represents an environmental challenge for disposal. However, OOME also contains some sugars (20–80 g/L), nitrogen compounds (12–24 g/L), organic acids (5–15 g/L), and residual oil (0.3–5 g/L). Mercadé et al. (1993) successfully used OOME for the production of RHL BS using the strain *Pseudomonas* sp. JAMM.

Oil cakes or soapstocks are semisolid or gummy product produced from processes oil seed where chemicals are used for extraction and refining the seed originated edible oils. The soapstock in spite of being a complex substrate has been successfully shown to produce highest yield of RHLs, along with different oily substrates, viz., sunflower oil, olive oil, soy bean oil. Yields up to 15.9 g/L were reported when using *P. aeruginosa* strain LBI grown in a salt medium containing soapstock (Benincasa et al., 2002). Soapstock has also been used efficiently for production of extracellular capsular polysaccharides (Benincasa et al., 2002)^.^ There have been examples of competent surfactant synthesis on soapstock and oil refinery wastes by *Candida antarctica* or *Candida apicola* with much higher yields than in the medium without addition of oil refinery waste (Bednarski et al., 2004). This shows the suitability of oil refinery waste for microbial surfactant production. Hydrophobic carbon sources like petroleum fractions, animal fat or vegetable oil have been utilized by several bacteria or yeast supplemented in cultured media for microbial surfactants (Hommel, 1990).

Two BEs namely, emulsan and biodispersan from *A*. *calcoaceticus* RAG-1 and *A*. *calcoaceticus* A2 were also produced by using soapstock as a carbon source (Shabtai, 1990). These two BEs show wide range of applications in stabilization of oil–water emulsion, the dispersion of large solid limestone granules and formation of micrometer-sized water suspension (Rosenberg and Ron, 1998). Soybean soapstock waste proved to be the best substrate with the yield of 11.7 g/L of RHLs that reduced the SFT in the culture broth to 26.9 mN/m with a critical micelle concentration (CMC) of 51.5 mg/L. Nitschke et al. (2005) reported production of mainly mono-RHL (RhaC_10_C_10_) when grown on hydrophobic substrates, while hydrophilic carbon sources lead to predominance of the di-RHL (RhaRhaC_10_C_10_) production. Pure soybean oil has been the predominant carbon source for many BSs production. Vollbrecht et al. (1999) tested similar oleic acid-rich oils, rapeseed oil and reported it to be efficient for BS production using *Tsukamurella* species DSM 44370. About 30 g/L glycolipid was produced from 110 g/L sunflower oil. The BS obtained showed high surface and interfacial activity and had some antimicrobial activities against some bacteria and a fungal strain.

### FOOD PROCESSING BY-PRODUCTS

Most of the edible oils, vegetable oils, saturated, unsaturated fats are used by food processing industries. Today the majority of food markets are dependent on these oils and fats. In addition to this, medicinal, pharmaceutical, cosmetic industries also use them. Once the oils are processed for food production, the residues become harmful pollutants to our environment. Wastes from soybean, potato, sweet potato, sweet sorghum contains high amount of starch that acts as base material in fermentation process. Waste products like canola meal, coconut cake, peanut cake, soybean cake, also represent suitable candidates for cheaper substrates (Mercadé et al., 1996). Processed olive oil, sunflower, ground nut oil, rape seed oil; potato peels are useful as raw material for microbial products. A peat, composed of decomposed vegetable matter contains high amount of carbohydrates with main sugars like glucose, galactose, xylose, and amino acids provides excellent source for the growth of microbes. Other by-products from vegetable oil refining processes are also becoming one of the most targeted substrates for microbial BS production process.

In addition to the above mentioned relatively cheap substrates a number of abundantly available starch base substrates provide another alternative renewable carbon sources. One of the representative examples is the potato processing industry that produces significant quantities of starch-rich waste substrates suitable for BS production. In addition to approximately 80% water contents, potato waste also has carbohydrates (17%), protein (2%), fat (0.1%), vitamins, inorganic minerals, and trace elements. Thus, potato wastes are a rich source of various components which can support the growth of microorganisms for production of various commercially important products. A commercially prepared potato starch in mineral salts medium was investigated by Fox and Bala (2000). They reported BS production by *B. subtilis* ATCC 21332 and a significant reduction in SFT from 71.3 to 28.3 mN/m with a CMC value of 0.10 g/L using a methylene chloride extract of the BS. Thompson et al. (2000), put forward the use of potato effluents containing high-solids (HSs) and low-solids (LSs) substrates for production of surfactin for a *Bacillus* strain. They used 10 time diluted effluents with or without trace minerals amendments and used corn steep liquor successfully to produce surfactin with slightly lower yields LS substrate than from optimized potato starch medium.

Thompson et al. (2001) also showed that the LSs potato effluents can be used for surfactin production after heat treatment without the need for complete sterilization and after pretreatments to enhance yields. Such studies are significant for finding applications in low-value applications like environmental remediation or oil recovery. Like molasses, sometimes potato based substrates also need to undergo pretreatment procedures involving heating, removal of starch particulates and acid hydrolysis. Thermal and acid pretreatment would help in the removal of contaminant vegetative cells yet can have mixed results on slight improvement or reduction in yields (Thompson et al., 2001). Other contributions were reported by Noah et al. (2002, 2005) where potato process effluents were used for production of BS from *B*. *subtilis* sp. in continuous culture and air left fermentation conditions. Noah et al. (2002) worked on improving the process for utilization of potato related substrate. However, they observed the yield of BS was restricted by the oxygen availability and competition for indigenous bacterial population. The same research group (Noah et al., 2005) carried out studies on surfactin production from *Bacillus* sp. by using purified potato starch and unamended LSs potato process eﬄuent. Their studies highlighted that the process is oxygen limited and that recalcitrant indigenous bacteria in the potato process eﬄuent hamper continuous surfactin production. They suggested the use of a chemostat operated in batch mode for surfactin production should be accomplished with the use of antifoam agents to prevent surfactant loss. They noted that antifoam does not interfere with recovery of surfactin and its efficacy and were able to achieve 0.6 g/L of surfactin from two different potato-processing facilities in comparison with Initial trials (0.9 g/L) from potato process eﬄuent. Thus, they established that cassava wastewater produced from the cassava flour preparation, a renewable inexpensive and easily available carbon source can be used for surfactin production by *B*. *subtilis* and other biotechnological processes. Different unconventional carbon sources such as potato peel powder, corn powder, sugarcane bagasse and *Madhuca indica* were also used by Jain et al. (2013). They reported increased viscosity in cultures yet achieved maximum SFT reduction when compared to other substrates. They reported an unidentified BS production at a yield of 15.40 ± 0.21 g/L on corn powder base production medium from *Klebsiella* sp. strain RJ-03 and concluded that the use of such cheap substrates have a significant potential for commercialization for applications in bioremediation processes.

## KINETICS OF BIOSURFACTANT PRODUCTION

It is well-established that along with environmental parameters such as pH, temperature, aeration, agitation, CO_2_ level etc.; BS production is also dependent on the substrate composition and concentration in the media. These parameters interact with each other in a complex way to affect the kinetics of the BS production. C:N ratio plays an important role in the production process. Nitrogen limitation has been reported to enhance production (Abu-Ruwaida et al., 1991). Patel and Desai (1997) reported that BS production was enhanced under nitrogen limiting conditions. Temperature, pH, aeration, and salt concentrations are of course important parameters that influence production at commercial levels (Navon-Venezia et al., 1995).

Silva et al. (2010) used *P. aeruginosa* UCP0992 to investigate the effect of both carbon and nitrogen (source and concentration) on BS production at different cultivation conditions such as aeration, temperature, and agitation speed. Growth and BS production in mineral medium formulated with 3% glycerol and 0.6% NaNO_3_, at 28°C during 120 h incubation at 200 rpm was monitored. They reported an almost parallel relationship between BS production, cell growth, consumption of glycerol, emulsification, SFT reduction, hexadecane, and other substrate utilization. They concluded that BS production is associated with growth starting shortly after inoculation with a two phase profile, the first up to 24 h and remaining constant until 48 h, while in the second phase, production increased at a slower rate up to 96 h with yields of 8.0 g/L. Biomass concentration was high (4.0 g/L) and glycerol consumption profile showed a similar pattern to SFT reduction, while, the hexadecane emulsification followed BS production. Such observations support the use of SFT and emulsification as indicative measures for the presence of BS molecules in the medium.

In studies carried out by Wei et al. (2005) on *P. aeruginosa* J4, isolated from wastewater of a petrochemical factory located in southern Taiwan, reported RHL production from different carbon substrates. Two complex media Luria Bertaini (LB) medium usually used as for *P. aeruginosa* strains and condensed molasses fermentation soluble (CMS) and a simpler glucose mineral salts (GMSs) medium were used to grow and produce RHL. RHL production was 1.7, 0.77 and 0.20 g/L on GMS, LB, and CMS media, respectively. It was also observed that high nitrogen content in a fermentation medium limits the BS production.

Wu et al. (2008) used an indigenous strain *P*. *aeruginosa* EM1 originating from an oil-contaminated site located in southern Taiwan to investigate RHLs improvement in GMS media by the response surface methodology. They changed carbon (glucose, sucrose, glycerol, olive oil, soybean oil, oleic acid, hexane) and inorganic nitrogen sources (NaNO_3_ and NH_4_Cl) and organic (yeast extract and urea). Maximum productivity of 136.4 and 71.8 mg/L/h was reported for glucose and glycerol, respectively. On the other hand, nitrate was the better inorganic nitrogen source (8.63 g/L) than ammonium ion (0.43 g/L) for RHL production. While organic sources were a very poor source of RHL production. The effect of C/N ratio on RHL production was thus investigated using two types of carbon sources (glucose or glycerol). The best RHL yield (6.8 g/L) occurred at a C/N ratio of 26 when glucose was used as the carbon source, whereas glycerol source yielded 7.5 g/L, at a C/N ratio of 52.

Literature surveys showed that, the Kinetics of biomass (BM), BS production, substrate utilization along with the fermentation duration required for growth of organisms are the most crucial parameters for commercial production processes. Raza et al. (2006) carried out kinetics for BS production for *P. aeruginosa* EBN-8 with different hydrocarbons viz., *n*-hexadecane paraffin oil, kerosene oil. Both *n*-hexadecane and paraffin oil, RHL production was 4.1 and 6.3 g/L respectively. Changing the carbon source and other parameter definitely affects the growth of organisms as well as the BS production.

Like *n*-hexadecane, other hydrocarbon namely *n*-octadecane 2% (v/v) has been proved to be supportive for the kinetic studies of BS production from *P. aeruginosa* OCD. Less than 5 days incubating conditions, in liquid Bushnell-Haas media with *n*-octadecane as the substrate resulted 0.98 mg/mL RHL in the culture broth at the stationary growth phase (Sahoo et al., 2011). Supplementation of multivalent cations viz., ZnSO_4_ followed by MnSO_4_ in the culture broth again, enhances the yield of BS production. This has been confirmed by authors thought monitoring the emulsification index assay.

Studies carried out by Khopade et al. (2012) included the kinetics of BS production up to 12 days for marine *Nocardiopsis* B4 under batch cultures condition. BSs from this halotolerant strains has potential for bioremediation of oil contaminated sites (oil spills). Investigation including measurement of SFT, emulsification assay, cell separation provides ample of information to understand the production of BS commercially.

Report available on probiotic bacteria like *L. pentosus* CECT-4023 has demonstrated strong BS production on cheese whey as an alternative medium. Carbon source viz., glucose, biomass and BS have been modeled according to reported models available in the literature (Rodrigues et al., 2006c). Their studies included four lactobacilli species for BS production by growing on De Man et al. (1960) MRS broth for *lactobacilli* strains as well as medium supplemented with whey. With MRS medium the yield of BS from *Lactobacillus casei* reached 1.6 g/L. For both *Lactobacillus rhamnosus* and *L. pentosus* BS yields were reported at 1.7 g/L and for *Lactobacillus coryniformis* subsp. *torquens* it was found to be 1.8 g/L. Further investigation of *L. pentosus* CECT-4023 showed BS production using whey as an alternative medium with low yield of 1.4 g/L. The growth of *L. pentosus* CECT-4023 is less on whey medium which may be probably due to the lack of some nutrients, although similar BS concentrations were obtained. The authors suggested that with a culture medium optimization it could be possible to achieve higher BS concentrations.

Pacheco et al. (2010) suggested that higher concentrations of glycerol, sodium nitrate, and yeast extract lead to increased yield of BS from *Rhodococcus erythropolis* strain ATCC 4277. The authors also revealed that increasing the phosphate buffer within the range between 60 and 150 mmol/L increases the yield of BS (285 mg/L) due to maintained pH during the fermentation process. Well-established methodologies have been proposed for BS production from *C. lipolytica* through the usage of soybean oil refinery residue (6%) and 1% glutamic acid supplementation. Rufino et al. (2014) explored growth-associated production of crude BS with a yield of 8.0 g/L from *C. lipolytica* UCP 0988 after 72 h of incubation. Sarubbo et al. (2007) worked on canola oil and glucose as cheaper substrates and documented 8.0 g/L of yield of BS produced by *C. lipolytica*. Other strain of *C. sphaerica* produced yields of 4.5 g/L at up to 144 h culture conditions. *C. sphaerica* has been reported for BS production of about 9 g/L after 144 h (Luna et al., 2011, 2012).

Factorial experimental design has been proved to be very supportive for studying the kinetics for production of microbial metabolites. Rocha et al. (2014) reported the production of BSs using cashew apple juice from *P. aeruginosa* MSIC02. They used 24 full factorial experimental design, using temperature, glucose concentration from cashew apple juice, phosphorous concentration and cultivation time as variables. Kinetics of growth and production of BSs by *P. aeruginosa* indicated reduction in SFT up to 47.7 to 28.0 dyn/cm and indicated production of surface active molecules.

In a recent molecular biology investigation (Perfumo et al., 2013) for the expression of the rhlB and rhlC rhamnosyltransferase genes responsible for RHLs production of *P*. *aeruginosa* strains showed no significant differences in the genes or the quantity or composition of RHLs congeners obtained by manipulating growth conditions. Fixed sequential expression patterns for rhlB and rhlC rhamnosyltransferase genes were observed during growth. They reported that it was not possible to induce significant up-regulation by varying producer strains or growth media. Their results indicated that the RHLs genes are highly conserved molecules and that their expression has a rather stringent control. The authors conclude that there is little opportunity to manipulate and greatly increase the yield of RHLs in *P. aeruginosa* strains. They also concluded that manipulating growth and medium composition conditions has little effect in the strains obtained from widely different environmental situations. In addition RHLs production was not greater on water-insoluble substrates than water-soluble ones, as often claimed in the past.

## FERMENTATION TECHNOLOGY: ROLE IN COMMERCIALIZATION OF BIOSURFACTANT PRODUCTION

When any industry is involved in the production of a particular compound, their main consideration is always achieving maximum profit from the minimum investment. BS industries are no exception to these policies and profitable applications are also of main concerns. The basic prerequisite of the BS/BE production industry is the type of substrate used in the production process. Since, some of the BS producing microbial communities are often isolated from oil, hydrocarbon-contaminated environments. Therefore, it is often assumed that water insoluble substrates are a necessity for the production of surface active agents. However, this fact may not always be true. Ample literature available suggests that water soluble carbon sources like glucose, fructose, sucrose etc., can be used in synthesizing the amphiphilic substances from a variety of microbial populations (Satpute et al., 2008, 2010a).

**FIGURE 3:**
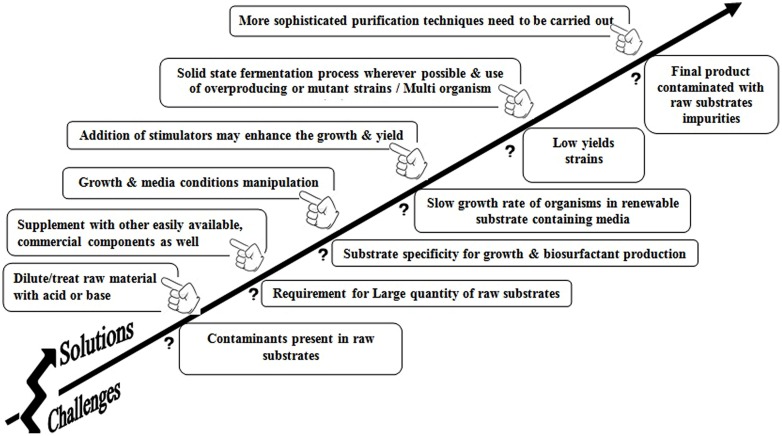
**Successful cram for increasing (he commercial yield of biosurfactant using renewable substrates**.

From an industrial point of view, using water soluble substrates is more attractive compared with using immiscible substrates. Therefore, the use of water soluble substrates particularly inexpensive industrial waste such as whey, molasses, distilleries eﬄuent, waste oils would help to bring down the production cost in industries. Subsequently, such efforts make BS fermentation technology feasible. However, there are certain advantages and disadvantages of using low-priced substrates for BS production as shown in **Table 4**. Several challenging problems and possible strategies to overcome these problems are represented in **Figure 3** to enhance the commercial yield of BS. It is also noted that though there are numerous reports and patents on BEs and BSs production, they are rarely used in commercial production process (Shete et al., 2006). One of the foremost reasons is the use of chemical based media for BS growth and production process. These exclusive chemicals enhance the production cost of these amphiphilic molecules. Very few attempts have been offered for the usage of renewable substrates. The second most important factor to consider is a cost effective separation/purification of amphiphilic substances. The purification procedures are significant in terms of time requirement and could account for up to 60% of the total production cost and may result in a low yield (Desai and Banat, 1997; Banat et al., 2010). Under such circumstance, the utilization of crude quality product or the direct fermentation broth with or without affecting the activity and potency of the actual product may be a solution. This fact has been well-supported from the studies carried out by Thavasi et al. (2011) who concluded that for environmental applications the BSs need not be pure and could be synthesized from a mixed cheaper carbon sources. It is possible to create an economically and environmentally viable mitigation technology for the bioremediation process. Noteworthy achievements in the field of genetic engineering technologies have promoted some significant advances such as the alteration in the substrate requirement of producing organisms. One of the best examples was reported by Koch et al. (1988) where insertion of *lac* plasmid from *E*. *coli* in *P. aeruginosa* was carried out for utilization of whey from dairy industry to produce BS.

**Table 4:** Advantages and disadvantages of cheaper substrates in biosurfactant production.

Advantages	Disadvantages
Commercial production cost can be reduced	Substrates contains undesired compounds
Many cheaper/renewable substrates are available	Processing or treatment of the substrates is required to use them as carbon, nitrogen, or energy source
Substrates are available in huge quantity	Final product itself get color or carry impurities from the substrates (e.g., molasses)
Enhanced the yield of biosurfactant/bioemulsifier	Special purification techniques needs to be employed to obtain the pure product, this increases the production of cost subsequently
Basic functional properties of the product do not change	Continuous supply of raw material with same composition may vary
Does not prove harmful to microorganisms	Raw substrates are may be very specific for different organisms
All components are eco-friendly and safe	A large quantity of raw substrates is essential, which may be difficult to get the continuous supply for the industrial process

The next consideration that may contribute to cost reduction is the duration of fermentations for some BS production. RHLs fermentations in most literature continue for up to 100 h while most of the production may have occurred in the initial 48 h. Prolonging the production time for a little more yield achievement may not be a cost effective undertaking particularly as most gene expression for RHLs production take place in the initial 24 h of fermentation (Perfumo et al., 2013). Routine use of cheap renewable substrates (agro-industrial wastes) and competent methods for recovery and purification of BSs can assist optimized conditions for high yields fermentation process on commercial scale. Another important aspect that should be highlighted here is biological remediation technologies used in the process on a larger extent. We can felicitate this process through developing innovative techniques such as foams or micro-foams (colloidal gas aphrons-CGA) in conjunction with BSs (Pacwa-Płociniczak et al., 2011). Some of the important criteria that need to be considered for production of surface active agents in industries are as follows:

• The type of substrate/raw materials
• Continuous supply of ingredients of same composition
• Potential microorganisms
• Purification process used for the recovery of surface active compounds
• Monetary inputs
• Marketing
• Application potential

The new exciting development in this current area of research and priorities to the interdisciplinary research approaches in combination with the technologies of large-scale fermentation and genetic engineering, BSs will be commercially successful compounds of the future (Saharan et al., 2011).

Our future work in the area of BS should be on the economics of BS production processes, particularly using the alternative low-cost fermentative media and reasonable cheaper product recovery process.

## CONCLUSION

Nature provides great immense possibilities for isolation of novel BEs or BSs producing microbial communities and products that can be utilized in the various application fields such as petroleum industry, detergents, pharmaceutical companies, agriculture, and personal health care products. The use of economically cheaper substrates is paving the way for cost effective BS production process in industries. Large scale production of these surface active compounds is promising; however, product with pure quality needs further streamlined approaches. Enormous data has been generated on application oriented properties like SFT, IFT, EA, wetting, foaming, detergency, and flocculation leading to wider applications in various industrial sectors. Use in bioremediation technology has received better treatment as hydrocarbon-polluted sites can be treated effectively with crude BSs products or the producing organisms. Although number of developments have taken place, it is important to note that BS production should be followed by minimum monetary input using cheap low cost waste materials while maintaining quality and quantity wherever possible. In future, our research on BS must be targeted on the economics of the fermentation processes of BS and BE, predominantly carried out through the practice of alternative low-cost effective production media and recovery processes.

## FUTURE PROSPECTS

Several food industries make use of various fats and oils that lead to the production of huge amount of high mass wastes, marine oils, soapstock, and free fatty acids from the extraction of seed oils. Searching for novel BS and BEs suitable for food industries has been steadily increasing and is expected to be a future prospect as more of these type molecules are included in food products. This is mainly driven by industries seeking to reduce dependency on plant emulsifiers produced by genetically modified crops. Other interesting areas include the use of BS producing microorganisms in composting. The most future potential application, however, are likely going to be related to oil industries application including bioremediation, cleaning, and microbial EOR both in oil sludge tank cleaning and oil well-recovery.

## Conflict of Interest Statement

The authors declare that the research was conducted in the absence of any commercial or financial relationships that could be construed as a potential conflict of interest.

## References

[B1] Abalos A. Pinazo A. Infante M. R. Casals M. Garcia F. Manresa A. (2001). Physicochemical and antimicrobial properties of new rhamnolipid produced by *Pseudomonas aeruginosa* AT10 from oil refinery wastes. *Langmuir* 1 1367–1371 10.1021/la0011735

[B2] Abouseoud M. Maachi R. Amrane A. (2007). Biosurfactant production from olive oil by *Pseudomonas fluorescens*. *Comm. Curr. Res. Educ. Top. Trends Appl. Microbiol.* 1 340–347.

[B3] Abu-Ruwaida A. S. Banat I. M. Haditirto S. Khamis A. (1991). Nutritional requirements and growth characteristics of a biosurfactant producing *Rhodococcus* bacterium. *World J. Microbiol. Biotechnol.* 7 53–61 10.1007/BF02310920 24424869

[B4] Alcantara V. A. Pajares I. G. Simbahan J. F. Rubio M. D. (2012). Substrate dependent production and isolation of an extracellular biosurfactant from *Saccharomyces cerevisiae* 2031. *Philipp. J. Sci.* 141 13–24.

[B5] Ashby R. D. Nunez A. Solaiman D. K. Y. Thomas A. F. (2005). Sophorolipid biosynthesis from a biodiesel coproduct stream. *J. Am. Oil Chem. Soc.* 9 625–630 10.1007/s11746-005-1120-3

[B6] Babu P. S. Vaidya A. N. Bal A. S. Kapur R. Juwarkar A. Khanna P. (1996). Kinetics of biosurfactant production by *Pseudomonas aeruginosa* strain BS2 from industrial wastes. *Biotechnol. Lett.* 18 263–268 10.1007/BF00142942

[B7] Banat I. M. Franzetti A. Gandolfi I. Bestetti G. Martinotti M. G. Fracchia L. (2010). Microbial biosurfactants production, applications and future potential. *Appl. Microbiol. Biotechnol.* 87 427–444 10.1007/s00253-010-2589-0 20424836

[B8] Banat I. M. Makkar R. S. Cameotra S. S. (2000). Potential commercial applications of microbial surfactants. *Appl. Microbiol. Biotechnol.* 53 495–508 10.1007/s002530051648 10855707

[B9] Bednarski W. Adamczak M. Tomasik J. Płaszczyk M. (2004). Application of oil refinery waste in the biosynthesis of glycolipids by yeast. *Bioresour. Technol.* 95 15–18 10.1016/j.biortech.2004.01.009 15207288

[B10] Benincasa M. (2007). Rhamnolipid produced from agroindustrial wastes enhances hydrocarbon biodegradation in contaminated soil. *Curr. Microbiol.* 54 445–449 10.1007/s00284-006-0610-8 17457644

[B11] Benincasa M. Abalos A. Oliveira I. Manresa A. (2004). Chemical structure, surface properties and biological activities of the biosurfactant produced by *Pseudomonas aeruginosa* LBI from soapstock. *Antonie Van Leeuwenhoek* 85 1–8 10.1023/B:ANTO.0000020148.45523.41 15028876

[B12] Benincasa M. Contiero J. Manresa M. A. Moraes I. O. (2002). Rhamnolipid production by *Pseudomonas aeruginosa* LBI growing on soapstock as the sole carbon source. *J. Food Eng*. 54 283–288 10.1016/S0260-8774(01)00214-X

[B13] Bento F. M. Gaylarde C. C. (1996). The production of interfacial emulsions by bacterial isolates from diesel. *Int. Biodeterior. Biodegrad.* 8 31–33 10.1016/S0964-8305(96)00021-2

[B14] Bhardwaj G. Cameotra S. S. Chopra H. K. (2013). Utilization of oleo-chemical industry by-products for biosurfactant production. *AMB Express* 3 68 10.1186/2191-0855-3-68 PMC3923564 24262384

[B15] Cameotra S. S. Makkar R. S. (2004). Recent applications of biosurfactants as biological and immunological molecules. *Curr. Opin. Microbiol.* 7 262–266 10.1016/j.mib.2004.04.006 15196493

[B16] Cammarota M. C. Freire D. M. G. (2006). A review on hydrolytic enzymes in the treatment of wastewater with high oil and grease content. *Bioresour. Technol.* 97 2195–2210 10.1016/j.biortech.2006.02.030 16621527

[B17] Cazetta M. L. Celligoi M. A. P. C. Buzato J. B. Scarmino I. S. Da Silva R. S. F. (2005). Optimization study for sorbitol production by *Zymomonas mobilis* in sugar cane molasses. *Process* *Biochem* 40 747–751 10.1016/j.procbio.2004.01.041

[B18] Chooklin C. S. Phertmean S. Cheirsilp B. Maneerat S. Saimmai A. (2013). Utilization of palm oil mill eﬄuent as a novel and promising substrate for biosurfactant production by *Nevskia ramosa* NA3. *Songklanakarin J. Sci. Technol.* 35 167–176; Accession** #** 89652747.

[B19] Cooper D. G. Paddock D. A. (1984). Production of a biosurfactant from *Torulopsis bombicola*. *Appl. Environ.* 47 173–176. 10.1128/aem.47.1.173-176.1984 PMC239631 16346455

[B20] Cunha C. D. Rosario M. Rosado A. S. Leite G. F. (2004). *Serratia* sp. SVGG16: a promising biosurfactant producer isolated from tropical soil during growth with ethanol-blended gasoline. *Process Biochem.* 39 2277–2282 10.1016/j.procbio.2003.11.027

[B21] Daniel H. J. Otto R. T. Binder M. Reus M. Syldatk C. (1999). Production of sophorolipids from whey: development of a two-stage process with *Cryptococcus curvatus* ATCC 20509 and *Candida bombicola* ATCC 22214 using deproteinized whey concentrates as substrates. *Appl. Microbiol. Biotechnol.* 51 40–45 10.1007/s002530051360 10077820

[B22] Daniel H. J. Reuss M. Syldatk C. (1998a). Production of sophorolipids in high concentration from deproteinized whey and rapeseed oil in a two stage fed batch process using *Candida bombicola* ATCC 22214 and *Cryptococcus curvatus* ATCC 20509. *Biotechnol. Lett.* 20 1153–1156 10.1023/A:1005332605003 10077820

[B23] Daniel H. J. Otto R. T. Reuss M. Syldatk C. (1998b). Sophorolipid production with high yields on whey concentrate and rapeseed oil without consumption of lactose. *Biotechnol. Lett.* 20 805–807 10.1023/B:BILE.0000015927.29348.1a

[B24] Daverey A. Pakshirajan K. Sangeetha P. (2009). Sophorolipids production by *Candida bombicola* using synthetic dairy wastewater. *World Acad. Sci. Eng. Technol.* 3 470–472 10.1007/s10098-010-0330-4

[B25] De Man J. C. Rogosa M. Sharpe M. E. (1960). A medium for cultivation of *Lactobacilli. J. Appl. Bacteriol.* 23 130–135 10.1111/j.1365-2672.1960.tb00188.x

[B26] Desai J. D. Banat I. M. (1997). Microbial production of surfactants and their commercial potential. *Microbiol. Mol. Rev.* 61 47–64. 10.1128/mmbr.61.1.47-64.1997 PMC232600 9106364

[B27] Deshpande M. Daniels L. (1995). Evaluation of sophorolipid biosurfactant production by *Candida bombicola* using animal fat. *Bioresour. Technol.* 54 143–150 10.1016/0960-8524(95)00116-6

[B28] Dubey K. V. Charde P. N. Meshram S. U. Yadav S. K. Singh S. Juwarkar A. (2012). Potential of new microbial isolates for biosurfactant production using combinations of distillery waste with other industrial wastes. *J. Pet. Environ. Biotechnol.* 12 1–11 10.4172/2157-7463.S1-002

[B29] Dubey K. Juwarkar A. (2001). Distillery and curd whey wastes as viable alternative sources for biosurfactant production. *World J. Microbiol. Biotechnol.* 17 61–69 10.1023/A:1016606509385

[B30] Dubey K. Juwarkar A. (2004). Determination of genetic basis for biosurfactant production in distillery and curd whey wastes utilizing *Pseudomonas aeruginosa* strain BS2. *Ind. J. Biotechnol.* 3 74–81.

[B31] Dubey K. V. Juwarkar A. A. Singh S. K. (2005). Bioseparations and downstream processing. Adsorption–desorption process using wood based activated carbon for recovery of biosurfactant from fermented) distillery wastewater. *Biotechnol. Prog.* 21 860–867 10.1021/bp040012e 15932266

[B32] Felse P. A. Shah V. Chan J. Rao K. J. Gross R. A. (2007). Sophorolipid biosynthesis by *Candida bombicola* from industrial fatty acid residues. *Enzyme Microb. Technol*. 40 316–323 10.1016/j.enzmictec.2006.04.013

[B33] Fiebig R. Detlef S. Jae-Chun C. Sung-Taik L. (1997). Biodegradation of polychlorinated biphenyls (PCBs) in the presence of a bioemulsifier produced on sunflower oil. *Biodegradation* 8 67–75 10.1023/A:1008256110136

[B34] Fiechter A. (1992). Biosurfactants: moving towards industrial application. *Trends Biotechnol.* 10 208–217 10.1016/0167-7799(92)90215-H 1368395

[B35] Fontes G. C. Ramos N. M. Amaral P. F. Nele F. M. Coelho M. A. Z. (2012). Renewable resources for biosurfactant production by *Yarrowia lipolytica. Braz. J. Chem. Eng.* 29 483–493 10.1590/S0104-66322012000300005

[B36] Fox S. L. Bala G. A. (2000). Production of surfactant from *Bacillus subtilis* ATCC 21332 using potato substrates. *Bioresour. Technol.* 75 235–240 10.1016/S0960-8524(00)00059-6

[B37] Fracchia L. Ceresa C. Franzetti A. Cavallo M. Gandolfi I. Van Hamme J. (2014). “Industrial applications of biosurfactants,” in *Biosurfactants: Production and Utilization—Processes, Technologies, and Economics* Chap. 12 eds Kosaric N. Sukan F. V. (Boca Raton: CRC Press), 245–260 10.1201/b17599-15

[B38] Franzetti A. Gandolfi I. Fracchia L. Van Hamme J. Gkorezis P. Marchant R. (2014). “Biosurfactant use in heavy metal removal from industrial effluents and contaminated sites,” in *Biosurfactants: Production and Utilization—Processes, Technologies, and Economics* Chap. 17 eds Kosaric N. Sukan F. V. (Boca Raton: CRC Press), 361–366 10.1201/b17599-20

[B39] Govindammal M. Parthasarathi R. (2013a). Biosurfactant production using pineapple juice as medium by *Pseudomonas fluorescens* isolated from mangrove forest soil. *Ind. Streams Res. J.* 2 1–10; Accession # : 87333604.

[B40] Govindammal M. Parthasarathi R. (2013b). Production and characterization of bio surfactant using renewable substrates by *Pseudomonas fluorescence* isolated from mangrove ecosystem. *J. Appl. Chem.* 2 55–62.

[B41] Guerra-Santos L. H. Kappeli O. Fiechter A. (1986). Dependence of *Pseudomonas aeruginosa* continuous culture biosurfactant production on nutritional and environmental factors. *Appl. Microbiol. Biotechnol.* 24 443–448 10.1007/BF00250320

[B42] Haba E. Espuny M. J. Busquets M. Manresa A. (2000). Screening and production of rhamnolipids by *Pseudomonas aeruginosa* 47T2 NCIB 40044 from waste frying oils. *J. Appl. Microbiol.* 88 379–387 10.1046/j.1365-2672.2000.00961.x 10747218

[B43] Helmy Q. Kardena E. Funamizu N. Wisjnuprapto. (2011). Strategies toward commercial scale of biosurfactant production as potential substitute for it’s chemically counterparts. *Int. J. Biotechnol.* 12 66–86 10.1504/IJBT.2011.042682

[B44] Hommel R. K. (1990). Formation and physiological role of biosurfactants produced by hydrocarbon-utilizing microorganisms. Biosurfactants in hydrocarbon utilization. *Biodegradation* 1 107–119 10.1007/BF00058830 1368144

[B45] Jain R. M. Mody K. Joshi N. Mishra A. Jha B. (2013). Effect of unconventional carbon sources on biosurfactant production and its application in bioremediation. *Int. J. Biol. Macromol.* 62 52–58 10.1016/j.ijbiomac.2013.08.030 23994788

[B46] Joshi S. Bharucha C. Jha S. Yadav S. Nerurkar A. Desai A. J. (2008). Biosurfactant production using molasses and whey under thermophilic conditions. *Bioresour. Technol.* 99 195–199 10.1016/j.biortech.2006.12.010 17321739

[B47] Kapadia S. G. Yagnik B. N. (2013). Current trend and potential for microbial biosurfactants. *Exp. Biol. Sci.* 4 1–8 10.12691/ijebb-2-2-4

[B48] Khopade A. Biao R. Liu X. Mahadik K. Zhang L. Kokare C. (2012). Production and stability studies of the biosurfactant isolated from marine *Nocardiopsis* sp. B4. *Desalination* 285 198–204 10.1016/j.desal.2011.10.002

[B49] Kim H. S. Jeon J. W. Kim B. H. Ahn C. Y. Mock O. H. H. Yoon B. D. (2006). Extracellular production of a glycolipid biosurfactant, mannosylerythritol lipid, by *Candida* sp. SY16 using fed-batch fermentation. *Appl. Microbiol. Biotechnol*. 70 391–396 10.1007/s00253-005-0092-9 16133323

[B50] Koch A. K. Raiser J. Kappeli O. Fiechter A. (1988). Genetic construction of lactose utilizing strains of *Pseudomona aeruginosa* and their application in biosurfactant production. *Nat. Biotechnol.* 6 1335–1339 10.1038/nbt1188-1335

[B51] Kosaric N. Cairns W. L. Gray N. C. C. Stechey D. Wood J. (1984). The role of nitrogen in multiorganism strategies for biosurfactant production. *J. Am. Oil Chem. Soc.* 61 1735–1743 10.1007/BF02582138

[B52] Lee S. Lee S. J. Kim S. Park I. Lee Y. Chung S. (2008). Characterization of new biosurfactant produced by *Klebsiella* sp. Y6-1 isolated from waste soybean oil. *Bioresour. Technol.* 99 2288–2292 10.1016/j.biortech.2007.05.020 17596933

[B53] Luna J. M. Rufino R. D. Albuquerque C. D. Sarubbo L. A. Campos-Takaki G. M. (2011). Economic optimized medium for tension-active agent production by *Candida sphaerica* UCP0995 and application in the removal of hydrophobic contaminant from sand. *Int. J. Mol. Sci.* 12 2463–2476 10.3390/ijms12042463 21731452 PMC3127128

[B54] Luna J. M. Rufino R. D. Campos-Takaki G. M. Sarubbo L. A. (2012). Properties of the biosurfactant produced by *Candida sphaerica* cultivated in low-cost substrates. *Chem. Eng. Trans.* 27 67–72 10.3303./CET12201

[B55] Makkar R. S. Cameotra S. S. (1997). Utilization of molasses for biosurfactant production by two *Bacillus strains* at thermophilic conditions. *J. Am. Oil Soc.* 74 887–889 10.1007/s11746-997-0233-7

[B56] Makkar R. S. Cameotra S. S. (1999). Biosurfactant production by microorganisms on unconventional carbon sources – a review. *J. Surfactants Deterg.* 2 237–241 10.1007/s11743-999-0078-3

[B57] Makkar R. S. Cameotra S. S. (2002). An update on uses of conventional substrates for biosurfactants production and their new applications. *Appl. Microbiol. Biotechnol.* 58 428–434 10.1007/s00253-001-0924-1 11954787

[B58] Makkar R. S. Cameotra S. S. Banat I. M. (2011). Advances in utilization of renewable substrates for biosurfactant production. *Appl. Microbiol. Biotechnol. Express* 1 1–19 10.1186/2191-0855-1-5 PMC3159906 21906330

[B59] Maneerat S. (2005). Production of biosurfactants using substrates from renewable resources. *Songklanakarin* *J. Sci. Technol*. 27 675–683.

[B60] Marchant R. Banat I. M. (2012a). Biosurfactants: a sustainable replacement for chemical surfactants? *Biotechnol. Lett.* 34 1597–1605 10.1007/s10529-012-0956-x 22618240

[B61] Marchant R. Banat I. M. (2012b). Microbial biosurfactants : challenges and opportunities for future exploitation. *Trends Biotechnol.* 30 558–565 10.1016/j.tibtech.2012.07.003 22901730

[B62] Marchant R. Funston S. Uzoigwe C. Rahman P. K. S. M. Banat I. M. (2014). “Production of biosurfactants from nonpathogenic bacteria,” in *Biosurfactants: Production and Utilization—Processes, Technologies, and Economics*, Chap. 5 eds Kosaric N. Sukan F. V. (Boca Raton: CRC Press) 73–82 10.1201/b17599-7

[B63] Meesters P. A. E. P. Huijberts G. N. M. Eggink G. (1996). High-cell-density cultivation of the lipid accumulating yeast *Cryptococcus curvatus* using glycerol as a carbon source. *Appl. Microbiol. Biotechnol*. 45 575–579 10.1007/s002530050731

[B64] Mercadé M. E. Manresa M. A. Robert M. Espuny M. J. Andrés C. Guinea J. (1993). Olive oil mill eﬄuent (OOME): new substrate for biosurfactant production. *Bioresour. Technol*. 43 1–6 10.1016/0960-8524(93)90074-L

[B65] Mercadé M. E. Monleon L. de Andres C. Rodon I. Martinez E. Espuny M. J. (1996). Screening and selection of surfactant- producing bacteria from waste lubricating oil. *J. Appl. Bacteriol.* 81 161–168 10.1111/j.1365-2672.1996.tb04494.x

[B66] Moldes A. B. Torrado A. M. Barral M. T. Domínguez J. M. (2007). Evaluation of biosurfactant production from various agricultural residues by *Lactobacillus pentosus*. *J. Agric. Food Chem.* 55 4481–4486 10.1021/jf063075g 17469840

[B67] Monteiro A. S. Coutinho J. Júnior A. C. Rosa C. A. Siqueira E. P. Santos V. L. (2009). Characterization of new biosurfactant produced by *Trichosporonm montevideense* CLOA 72 isolated from dairy industry effluents. *J. Basic Microbiol.* 49 1–11 10.1002/jobm.200900089 19810042

[B68] Monteiro A. S. Domingues V. S. Souza M. V. Lula I. Gonçalves D. B. de Siqueira E. P. (2012). Bioconversion of biodiesel refinery waste in the bioemulsifier by *Trichosporon mycotoxinivorans* CLA2. *Biotechnol. Biofuels* 5 1–12. 10.1186/1754-6834-5-29 22559210 PMC3485625

[B69] Morita T. Konish M. Fukuoka T. Imura T. Kitamoto D. (2007). Microbial conversion of glycerol into glycolipid biosurfactants, mannosylerythritol lipids, by a basidiomycete yeast, *Pseudozyma antarctica* JCM 10317. *J. Biosci. Bioeng.* 104 78–81 10.1263/jbb.104.78 17697987

[B70] Muriel J. M. Bruqu J. M. Olfas J. M. Jimenez-Sanchez A. (1996). Production of biosurfactant by *Cladosporium resinae*. *Biotechnol. Lett*. 18 235–240 10.1007/BF00142937

[B71] Navon-Venezia S. Zosim Z. Gottlieb A. Legmann R. Carmeli S. Ron E. Z. (1995). Alasan, a new bioemulsifier from *Acinetobacter radioresistens*. *Appl. Environ. Microbiol.* 61 3240–3244. 7574633 10.1128/aem.61.9.3240-3244.1995 PMC167603

[B72] Nitschke M. Costa S. G. Contiero J. (2010). Structure and applications of a rhamnolipid surfactant produced in soybean oil waste. *Appl. Biochem. Biotechnol*. 160 2066–2074 10.1007/s12010-009-8707-8 19649781

[B73] Nitschke M. Costa S. G. Haddad R. Gonc L. A. G. Alves N. C. Eberlin M. N. (2005). Oil wastes as unconventional substrates for rhamnolipid biosurfactant production by *Pseudomonas aeruginosa* LBI. *Biotechnol. Prog.* 21 1562–1566 10.1021/bp050198x 16209563

[B74] Nitschke M. Ferraz C. Pastore G. M. (2004). Selection of microorganisms for biosurfactant production using agroindustrial wastes. *Braz. J. Microbiol.* 35 81–85 10.1590/S1517-83822004000100013

[B75] Nitschke M. Pastore G. M. (2004). Biosurfactant production by *Bacillus subtilis* using cassava processing eﬄuent. *Appl. Biochem. Biotechnol*. 112 163–172 10.1385/ABAB:112:3:163 15007184

[B76] Nitschke M. Pastore G. M. (2006). Production and properties of a surfactant obtained from *Bacillus subtilis* grown on cassava wastewater. *Bioresour. Technol.* 97 336–341 10.1016/j.biortech.2005.02.044 16171690

[B77] Noah K. S. Bruhn D. F. Bala G. A. (2005). Surfactin production from potato process eﬄuent by *Bacillus subtilis* in a chemostat. *Appl. Biochem. Biotechnol.* 122 465–474 10.1385/ABAB:122:1-3:0465 15920256

[B78] Noah K. S. Sandra L. F. Debby F. B. David N. T. Gregory A. B. (2002). Development of continuous surfactin production from potato process eﬄuent by *Bacillus subtilis* in an airlift reactor. *Appl. Biochem. Biotechnol*. 98–100, 803–813 10.1385/ABAB:98-100:1-9:803 12018303

[B79] Ohno A. Takashi A. Shoda M. (1992). Production of a lipopetide antibiotic surfactin by recombinant *Bacillus subtilis* NB22 using wheat bran as substrate. *Biotechnol. Lett.* 14 817–822.

[B80] Ohno A. Takashi A. Shoda M. (1993). Production of antifungal peptide antibiotic iturin by *Bacillus subtilis* NB22 in solid state fermentation. *J. Ferment. Bioeng.* 75 23–27 10.1016/0922-338X(93)90172-5

[B81] Ohno A. Takashi A. Shoda M. (1995). Production of lipopeptide antibiotic surfactin by recombinant *Bacillus subtilis* in solid state fermentation. *Biotechnol. Bioeng.* 47 209–214 10.1002/bit.260470212 18623394

[B82] Oliveira F. J. S. Vazquez L. De Campos N. P. de França F. P. (2006). Biosurfactants production by *Pseudomonas aeruginosa* FR using palm oil. *Appl. Biochem. Biotehnol.* 131 727–737 10.1385/ABAB:131:1:727 18563649

[B83] Onbasli D. Aslim B. (2009). Biosurfactant production in sugar beet molasses by some *Pseudomonas* spp. *J. Environ. Biol.* 30 161–163. 20112880

[B84] Otto R. T. Daniel H. J. Pekin G. Müller-Decker K. Fürstenberger G. Reuss M. (1999). Production of sophorolipids from whey. II. Product composition, surface active properties, cytotoxicity and stability against hydrolases by enzymatic treatment. *Appl. Microbiol. Biotechnol.* 52 495–501; Accession # WOS:000083529900006 10.1007/s002530051551 10570796

[B85] Pacheco C. J. Ciapina E. M. P. Gomes E. D. B. Pereira N. Jr. (2010). Biosurfactant production by *Rhodococcus erythropolis* and its application to oil removal. *Braz. J. Microbiol.* 41 685–693 10.1590/S1517-83822010000300019 24031544 PMC3768661

[B86] Pacwa-Płociniczak M. Płaza G. A. Piotrowska-Seget Z. Cameotra S. S. (2011). Environmental applications of biosurfactants: recent advances. *Int. J. Mol. Sci.* 12 633–654 10.3390/ijms12010633 21340005 PMC3039971

[B87] Pansiripata S. Pornsunthorntaweea O. Rujiravanita R. Kitiyanana B. Somboonthanatea P. Chavadeja S. (2010). Biosurfactant production by *Pseudomonas aeruginosa* SP4 using sequencing batch reactors: effect of oil-to-glucose ratio. *Biochem. Eng. J.* 49 185–191 10.1016/j.bej.2009.12.011

[B88] Patel R. M. Desai A. J. (1997). Biosurfactant production by *Pseudomonas aeruginosa* GS3 from molasses. *Lett. Appl. Microbiol*. 25 91–94 10.1046/j.1472-765X.1997.00172.x

[B89] Patricia B. Jean-Claude B. (1999). Involvement of bioemulsifier in heptadecane uptake in *Pseudomonas nautica*. *Chemosphere* 38 1157–1164 10.1016/S0045-6535(98)00366-X 10028664

[B90] Pekin G. Vardar-Sukan E. Kosaric N. (2005). Production of sophorolipids from *Candida bombicola* ATCC 22214 using Turkish corn oil and honey. *Eng. Life Sci.* 5 357–362 10.1002/elsc.200520086

[B91] Perfumo A. Rudden M. Smyth T. J. P. Marchant R. Stevenson P. S. Parry N. J. (2013). Rhamnolipids are conserved biosurfactants molecules: implications for their biotechnological potential. *Appl. Microbiol. Biotechnol.* 97 7297–7306 10.1007/s00253-013-4876-z 23563913

[B92] Prabhu Y. Phale P. S. (2003). Biodegradation of phenanthrene by *Pseudomonas* sp. strain PP2: novel metabolic pathway, role of biosurfactant and cell surface hydrophobicity in hydrocarbon assimilation. *Appl. Microbiol. Biotechnol*. 61 342–351 10.1007/s00253-002-1218-y 12743764

[B93] Rahman K. S. M. Rahman T. J. McClean S. Marchant R. Banat I. M. (2002). Rhamnolipid biosurfactant production by strains of *Pseudomonas aeruginosa* using low- cost materials. *Biotechnol. Prog.* 18 1277–1281 10.1021/bp020071x 12467462

[B94] Rashedi H. Assadi M. M. Bonakdarpour B. Jamshidi E. (2005a). Environmental importance of rhamnolipid production from molasses as a carbon source. *Int. J. Environ. Sci. Technol.* 2 59–62 10.1007/BF03325858

[B95] Rashedi H. Jamshidi E. Assadi M. M. Bonakdarpour B. (2005b). Isolation and production of biosurfactant from *Pseudomonas aeruginosa* isolated from Iranian southern wells oil. *Int. J. Environ. Sci. Technol.* 2 121–127.

[B96] Rashedi H. Mazaheri Assadi M. Jamshidi E. Bonakdarpour B. (2006). Production of rhamnolipids by *Pseudomonas aeruginosa* growing on carbon sources. *Int. J. Enviorn. Sci. Technol.* 3 297–303 10.1007/BF03325937

[B97] Raza Z. A. Khan M. S. Khalid Z. M. (2007a). Physicochemical and surface-active properties of biosurfactant produced using molasses by a *Pseudomonas aeruginosa* mutant. *J. Environ. Sci. Health A Tox. Hazard. Subst. Environ. Eng.* 42 73–80 10.1080/10934520601015784 17129951

[B98] Raza Z. A. Rehman A. Khan M. S. Khalid Z. M. (2007b). Improved production of biosurfactant by a *Pseudomonas aeruginosa* mutant using vegetable oil refinery wastes. *Biodegradation* 18 115–121 10.1007/s10532-006-9047-9 16491304

[B99] Raza Z. A. Khana M. S. Khalidb Z. M. Rehmanb A. (2006). Production of biosurfactant using different hydrocarbons by *Pseudomonas aeruginosa* EBN-8 Mutant. *Z. Naturforsch.* C 61 87–94. 10.1515/znc-2006-1-216 16610223

[B100] Robert M. Mercade M. E. Bosch M. P. Parra J. L. Espuny M. J. Manresa M. A. (1989) Effect of carbon source on biosurfactant production by *Pseudomonas aeruginosa* 44T1. *Biotechnol. Lett.* 11 871–874 10.1007/BF01026843

[B101] Rocha M. V. P. Mendes J. S. Qiro M. E. A. Melo V. M. M. Rocha L. Gonçalves B. (2014). Biosurfactant production by *Pseudomonas aeruginosa* MSIC 02 in cashew apple juice using a 24 full factorial experimental design *Chem. Ind. Chem. Eng. Q.* 20 49-58. 10.2298/CICEQ120518100R

[B102] Rodrigues L. Banat I. M. Teixeira J. Oliveira R. (2006a). Biosurfactants: potential applications in medicine. *J. Antimicrob. Chemother.* 57 609–618 10.1093/jac/dkl024 16469849

[B103] Rodrigues L. R. Teixeira J. A. Oliveira R. (2006b). Low-cost fermentative medium for biosurfactant production by probiotic bacteria. *Biochem. Eng. J.* 32 135–142 10.1016/j.bej.2006.09.012

[B104] Rodrigues L. Moldes A. Teixeira J. Oliveira R. (2006c). Kinetic study of fermentative biosurfactant production by *Lactobacillus* strains. *Biochem. Eng. J.* 28 109–116 10.1016/j.bej.2005.06.001

[B105] Rodrigues L. R. Teixeira J. A. (2008). “Biosurfactants production from cheese whey,” in *Advances in Cheese Whey Utilization* Vol. 8 eds Cerd’an M. E. Gonz’alez-Siso M. Becerra M. (Trivandrum: India-Transworld Research Network) 81–104.

[B106] Rosenberg E. Ron E. Z. (1998). “Surface active polymers from the genus *Acinetobacter*,” in *Biopolymers from Renewable Resources* ed. Kaplan D. L. (Berlin: Springer), 281–291 10.1007/978-3-662-03680-8_11

[B107] Rufino R. D. Luna J. M. de Campos Takaki G. M. Sarubbo L. A. (2014). Characterization and properties of the biosurfactant produced by *Candida lipolytica* UCP 0988. *Electron. J. Biotechnol.* 17 34–38 10.1016/j.ejbt.2013.12.006

[B108] Rufino R. D. Sarubbo L. A. Campos-Takaki G. M. (2007). Enhancement of stability of biosurfactant produced by *Candida lipolytica* using industrial residue as substrate. *World J. Microbiol. Biotechnol*. 23 729–734 10.1007/s11274-006-9278-2

[B109] Saharan B. S. Sahu R. K. Sharma D. (2011). A review on biosurfactants: fermentation, current developments and perspectives. *Genet. Eng. Biotechnol. J.* 29 1–14.

[B110] Sahoo S. Datta S. Biswas D. (2011). Optimization of culture conditions for biosurfactant production from *Pseudomonas aeruginosa* OCD1. *J. Adv. Sci. Res.* 2 32–36.

[B111] Saimmai A. Onlamool T. Sobhon V. Maneerat S. (2012). “Diversity of biosurfactants/bioemulsifiers-producing bacteria isolated from palm oil contaminated soils in palm oil industry,” in *Proceedings of the 38th Congress on Science and Technology of Thailand “Science for the Future of Mankind”* (Chiang Mai: The Empress Convention Hall) 1–6.

[B112] Santa Anna L. M. Sebastian G. V. Menezes E. P. Alves T. L. M. Santos A. S. Pereira N. Jr. (2002). Production of biosurfactants from *Pseudomonas aeruginosa* PA1 isolated in oil environments. *Braz. J. Chem. Eng.* 19 159–166 10.1590/S0104-66322002000200011

[B113] Santos D. K. F. Rufino R. D. Luna J. M. Santos V. A. Salgueiro A. A. Sarubbo L. A. (2013). Synthesis and evaluation of biosurfactant produced by *Candida lipolytica* using animal fat and corn steep liquor. *J. Pet. Sci. Eng.* 105 43–50 10.1016/j.petrol.2013.03.028

[B114] Saravanan V. Subramaniyan V. (2014). Production of biosurfactant by *Pseudomonas aeruginosa* PB3A using agro-industrial wastes as a carbon source. *Malays. J. Microbiol.* 10 57–62.

[B115] Sarubbo L. A. Farias C. B. B. Campos-Takaki G. M. (2007). Co-utilization of canola oil and glucose on the production of a surfactant by *Candida lipolytica*. *Curr. Microbiol.* 54 68–73 10.1007/s00284-006-0412-z 17171462

[B116] Sarubbo L. A. Marcal M. C. R. Campos-Takaki G. M. (1997). Comparative-study on bioemulsifiers produced by *Candida lipolytica* strains. *Arquivos Biol. E Tecnol.* 40 707–720.

[B117] Sarubbo L. A. Porto A. L. F. Campos-Takaki G. M. (1999). The use of babassu oil as substrate to produce bioemulsifiers by *Candida lipolytica. Can. J. Microbiol.* 45 423–426 10.1139/w99-025 10446719

[B118] Satpute S. K. Banat I. M. Dhakephalkar P. K. Banpurkar A. G. Chopade B. A. (2010a). Biosurfactants, bioemulsifiers and exopolysaccharides from marine microorganisms. *Biotechnol. Adv.* 28 436–450 10.1016/j.biotechadv.2010.02.006 20172021

[B119] Satpute S. K. Banpurkar A. G. Dhakephalkar P. K. Banat I. M. Chopade B. A. (2010b). Methods for investigating biosurfactants and bioemulsifiers: a review. *Crit. Rev. Biotechnol.* 30 127–144 10.3109/07388550903427280 20210700

[B120] Satpute S. K. Bhuyan S. S. Pardesi K. R. Mujumdar S. S. Dhakephalkar P. K. Shete A. M. (2010c). “Molecular genetics of biosurfactants and bioemulsifiers synthesis in microorganisms,” in *Biosurfactants* ed. Sen R. (New York, NY: Springer Science+Business meida, LCC Landes Bioscience) 15–33.

[B121] Satpute S. K. Bhawsar B. D. Dhakephalkar P. K. Chopade B. A. (2008). Assessment of different screening methods for selecting biosurfactant producing marine bacteria. *Ind. J. Mar. Sci*. 37 243–250.

[B122] Shabtai Y. (1990). Production of exopolysaccharides by *Acinetobacter* strains in a controlled fed-batch fermentation process using soap stock oil (SSO) as carbon source. *Int. J. Biol. Macromol.* 12 145–152 10.1016/0141-8130(90)90066-J 2078530

[B123] Sheppard J. D. Mulligan N. C. (1987). The production of surfactin by *Bacillus subtilis* grown on peat hydrolysate. *Appl. Microbiol. Biotechnol.* 27 110–116 10.1007/BF00251931

[B124] Shete A. M. Wadhawa G. Banat I. M. Chopade B. A. (2006). Mapping of patents on bioemulsifier and biosurfactant: a review. *J. Sci. Ind. Res.* 65 91–115.

[B125] Shreve G. S. Inguva S. Gunnam S. (1995). Rhamnolipid biosurfactant enhancement of hexadecane biodegradation by *Pseudomonas aeruginosa*. *Mol. Mar. Biol. Biotechnol*. 4 331–337 10.1128/AEM.68.9.4502-4508.2002 8541984

[B126] Silva S. N. Farias C. B. Rufino R. D. Luna J. M. Sarubbo L. A. (2010). Glycerol as substrate for the production of biosurfactant by *Pseudomonas aeruginosa* UCP0992. *Colloids Surf. B Biointerfaces* 79 174–183 10.1016/j.colsurfb.2010.03.050 20417068

[B127] Smyth T. J. P. Perfumo A. Marchant R. Banat I. M. (2010a). “Isolation and analysis of low molecular weight microbial glycolipids,” in *Handbook of Hydrocarbon and Lipid Microbiology* Part 2 Chap. 28 ed. Timmis K. N. (Berlin Heidelberg: Springer-Verlag) 3705–3723 10.1007/978-3-540-77587-4_291

[B128] Smyth T. J. P. Perfumo A. Mcclean S. Marchant R. Banat I. M. (2010b). “Isolation and analysis of lipopeptides and high molecular weight biosurfactants,” in *Handbook of Hydrocarbon and Lipid Microbiology* Chap. 27 ed. Timmis K. N. (Berlin Heidelberg: Springer-Verlag), 3689–3704 10.1007/978-3-540-77587-4_290

[B129] Solaiman D. K. Y. Ashby R. D. Foglia T. A. Nuñez A. Marmer W. N. (2003). “Fermentation-based processes for the conversion of fats, oils and derivatives into biopolymers and biosurfactants,” in *Proceedings of the 31st United States-Japan Cooperative Program in Natural Resources (UJNR), Protein Resources Panel* (Tsukuba: Eastern Regional Research Center, ARS, USDA), V1–V10.

[B130] Solaiman D. K. Y. Ashby R. D. Nunez A. Foglia T. A. (2004). Production of sophorolipids by *Candida bombicola* grown on soy molasses as substrate. *Biotechnol. Lett.* 26 1241–1245 10.1023/B:BILE.0000036605.80577.30 15289681

[B131] Solaiman D. K. Y. Ashby R. D. Zerkowski J. A. Foglia T. A. (2007). Simplified soy molasses-based medium for reduced-cost production of sophorolipids by *Candida bombicola*. *Biotechnol. Lett.* 29 1341–1347 10.1007/s10529-007-9407-5 17541506

[B132] Thaniyavarn J. Chianguthai T. Sangvanich P. Roongsawang N. Washio K. Morikawa M. (2008). Production of sophorolipid production by *Pichia* anomala. *Basic Biotechnol. Biochem*. 72 2061–2068 10.1271/bbb.80166 18685212

[B133] Thanomsub B. Watcharachaipong T. Chotelersak K. Arunrattiyakorn P. Nitoda T. Kanzaki H. (2004). Monoacylglycerols glycolipid biosurfactants produced by a thermotolerant yeast, *Candida ishiwadae*. *J. Appl. Microbiol*. 96 588–592 10.1111/j.1365-2672.2004.02202.x 14962139

[B134] Thavasi R. Jayalakshmi S. Balasubramanian T. Banat I. M. (2007). Biosurfactant production by *Corynebacterium kutscheri* from waste motor lubricant oil and peanut oil cake. *Lett. Appl. Microbiol.* 45 686–691 10.1111/j.1472-765X.2007.02256.x 17944837

[B135] Thavasi R. Jayalakshmi S. Banat I. M. (2011). Application of biosurfactant produced from peanut oil cake by *Lactobacillus delbrueckii* in biodegradation of crude oil. *Bioresour. Technol.* 102 3366–3372 10.1016/j.biortech.2010.11.071 21144745

[B136] Thavasi R. Marchant R. Banat I. M. (2014). “Biosurfactant applications in agriculture,” in *Biosurfactants: Production and Utilization—Processes, Technologies, and Economics* Chap. 15 eds Kosaric N. Sukan F. V. (New York, NY: CRC Press) 313–326 10.1201/b17599-18

[B137] Thavasi R. Subramanyam Nambaru V. R. M. Jayalakshmi S. Balasubramanian T. Banat I. M. (2009). Biosurfactant production by *Azotobacter chroococcum* isolated from the marine environment. *Mar. Biotechnol.* (N. Y.) 11 551–556 10.1007/s10126-008-9162-1 19034398

[B138] Thompson D. N. Fox S. L. Bala G. A. (2000). Biosurfactants from potato process effluents. *Appl. Biochem. Biotechnol.* 84–86, 917–930 10.1385/ABAB:84-86:1-9:917 10849846

[B139] Thompson D. N. Fox S. L. Bala G. A. (2001). The effects of pretreatments on surfactin production from potato process eﬄuent by *Bacillus subtilis*. *Appl. Biochem. Biotechnol.* 91–93, 487–502 10.1385/ABAB:91-93:1-9:487 11963877

[B140] Trummler K. Effenberger F. Syldatk C. (2003). An integrated microbial/enzymatic process for production of rhamnolipids and L-(+)-rhamnose from rapeseed oil with *Pseudomonas* sp. DSM 2874. *Eur. J. Lipid Sci. Technol.* 105 563–571 10.1002/ejlt.200300816

[B141] Vance-Harrop M. H. de Gusmão N. B. de Campos-Takaki G. M. (2003). New bioemulsifiers produced by *Candida lipolytica* using d-glucose and babassu oil as carbon sources. *Braz. J. Microbiol.* 34 120–123 10.1590/S1517-83822003000200006

[B142] Vollbrecht E. Rau U. Lang S. (1999). Microbial conversion of vegetable oils surface active di-, tri-, and tetrasaccharide lipids (biosurfactants) by the bacterial strain *Tsukamurella* spec. *Fett/Lipid* 101 389–394 10.1002/(SICI)1521-4133(199910)101:103.0.CO;2-9

[B143] Wei Y.-H. Chou C. L. Chang J. S. (2005). Rhamnolipid2 production by indigenous *Pseudomonas aeruginosa* J4 originating from petrochemical wastewater. *Biochem. Eng. J.* 27 146–154 10.1016/j.bej.2005.08.028

[B144] Willey R. (2001). Fats, oils and greases: the minimization and treatment of wastewaters generated from oil refining and margarine production. *Ecotoxicol. Environ. Saf.* 50 127–133 10.1006/eesa.2001.2081 11689028

[B145] Wu J. Y. Yeh K. L. Lu W. B. Lin C. L. Chang J. S. (2008). Rhamnolipid production with indigenous *Pseudomonas aeruginosa* EM1 isolated from oil-contaminated site. *Bioresour. Technol.* 99 1157–1164 10.1016/j.biortech.2007.02.026 17434729

[B146] Zhou Q. H. Kosaric N. (1993). Effect of lactose and olive oil on intra-and extracellular lipids of *Torulopsis bombicola*. *Biotechnol. Lett.* 15 477–482 10.1007/BF00129322

[B147] Zhou Q. H. Kosaric N. (1995). Utilization of canola oil and lactose to produce biosurfactant with *Candida bombicola*. *J. Am. Oil Chem. Soc.* 72 67–71 10.1007/BF02635781

[B148] Zhou Q. H. Klekner V. Kosaric N. (1992). Production of sophorose lipids by *Torulopsis bombicola* from safflower oil and glucose. *J. Am. Oil Chem. Soc.* 69 89–91 10.1007/BF02635883

